# Mitochondrial single-stranded DNA binding protein novel *de novo SSBP1* mutation in a child with single large-scale mtDNA deletion (SLSMD) clinically manifesting as Pearson, Kearns-Sayre, and Leigh syndromes

**DOI:** 10.1371/journal.pone.0221829

**Published:** 2019-09-03

**Authors:** Margaret A. Gustafson, Elizabeth M. McCormick, Lalith Perera, Matthew J. Longley, Renkui Bai, Jianping Kong, Matthew Dulik, Lishuang Shen, Amy C. Goldstein, Shana E. McCormack, Benjamin L. Laskin, Bart P. Leroy, Xilma R. Ortiz-Gonzalez, Meredith G. Ellington, William C. Copeland, Marni J. Falk

**Affiliations:** 1 Genome Integrity and Structural Biology Laboratory, NIEHS, NIH, Research Triangle Park, NC, United States of America; 2 Mitochondrial Medicine Frontier Program, Division of Human Genetics, Department of Pediatrics, Children’s Hospital of Philadelphia, Philadelphia, PA, United States of America; 3 GeneDx, Gaithersburg, MD, United States of America; 4 Department of Pathology and Laboratory Medicine, Children’s Hospital of Philadelphia, Philadelphia, PA, United States of America; 5 Center for Personalized Medicine, Department of Pathology & Laboratory Medicine, Children’s Hospital Los Angeles, Los Angeles, CA, United States of America; 6 Department of Pediatrics, University of Pennsylvania Perelman School of Medicine, Philadelphia, PA, United States of America; 7 Division of Endocrinology and Diabetes, Children's Hospital of Philadelphia, Philadelphia, PA, United States of America; 8 Division of Nephrology, Children's Hospital of Philadelphia, Philadelphia, PA, United States of America; 9 Center for Medical Genetics Ghent, Ghent University and Ghent University Hospital, Ghent, Belgium; 10 Department of Ophthalmology, Ghent University Hospital, Ghent, Belgium; 11 Division of Ophthalmology, Children's Hospital of Philadelphia, Philadelphia, PA, United States of America; 12 Division of Neurology, Children's Hospital of Philadelphia, Philadelphia, PA, United States of America; Ben-Gurion University of the Negev, ISRAEL

## Abstract

Mitochondrial DNA (mtDNA) genome integrity is essential for proper mitochondrial respiratory chain function to generate cellular energy. Nuclear genes encode several proteins that function at the mtDNA replication fork, including mitochondrial single-stranded DNA-binding protein (SSBP1), which is a tetrameric protein that binds and protects single-stranded mtDNA (ssDNA). Recently, two studies have reported pathogenic variants in SSBP1 associated with hearing loss, optic atrophy, and retinal degeneration. Here, we report a 14-year-old Chinese boy with severe and progressive mitochondrial disease manifestations across the full Pearson, Kearns-Sayre, and Leigh syndromes spectrum, including infantile anemia and bone marrow failure, growth failure, ptosis, ophthalmoplegia, ataxia, severe retinal dystrophy of the rod-cone type, sensorineural hearing loss, chronic kidney disease, multiple endocrine deficiencies, and metabolic strokes. mtDNA genome sequencing identified a single large-scale 5 kilobase mtDNA deletion (m.8629_14068del5440), present at 68% and 16% heteroplasmy in the proband’s fibroblast cell line and blood, respectively, suggestive of a mtDNA maintenance defect. On trio whole exome blood sequencing, the proband was found to harbor a novel *de novo* heterozygous mutation c.79G>A (p.E27K) in *SSBP1*. Size exclusion chromatography of p.E27K SSBP1 revealed it remains a stable tetramer. However, differential scanning fluorimetry demonstrated p.E27K SSBP1 relative to wild type had modestly decreased thermostability. Functional assays also revealed p.E27K SSBP1 had altered DNA binding. Molecular modeling of SSBP1 tetramers with varying combinations of mutant subunits predicted general changes in surface accessible charges, strength of inter-subunit interactions, and protein dynamics. Overall, the observed changes in protein dynamics and DNA binding behavior suggest that p.E27K SSBP1 can interfere with DNA replication and precipitate the introduction of large-scale mtDNA deletions. Thus, a single large-scale mtDNA deletion (SLSMD) with manifestations across the clinical spectrum of Pearson, Kearns-Sayre, and Leigh syndromes may result from a nuclear gene disorder disrupting mitochondrial DNA replication.

## Introduction

Mitochondrial disease is highly heterogeneous in etiology, with more than 350 causal genes across both nuclear and mitochondrial genomes[1]. Mitochondrial DNA (mtDNA) disorders can include point mutations, as well as single or multiple large-scale mtDNA deletions and duplications. Whereas multiple large mtDNA deletions are widely recognized to result from pathogenic variants involving a wide array of possible nuclear gene disorders[2], single large-scale mtDNA deletions (SLSMDs) have long been thought to occur sporadically in affected individuals or oocytes, since they were first discovered to cause human disease 30 years ago[3]. Furthermore, SLSMDs have classically been thought to manifest as one of several discrete clinical syndromes that present in different individuals at specific ages. These classical clinical syndromes include: (1) Pearson syndrome, which presents with infantile sideroblastic anemia and exocrine pancreatic insufficiency; (2) Kearns-Sayre syndrome (KSS), which generally presents in the second decade with progressive multi-system disease including retinal dystrophy, progressive external ophthalmoplegia (PEO) and a range of other possible features including ptosis, cerebellar ataxia, intellectual disability, dementia, sensorineural hearing loss, cardiac conduction block with sudden death, gastrointestinal dysmotility, endocrinopathies, muscle weakness, and exercise intolerance; and (3) Chronic progressive external ophthalmoplegia (CPEO), which manifests in adults with eye movement paralysis (ophthalmoplegia), ptosis, oropharyngeal weakness, and proximal myopathy with exercise intolerance[4]. Leigh syndrome involving progressive subacute necrotizing encephalomyopathy, regression with illness, and a range of neurologic disabilities has also been seen in those with a SLSMD, although it manifests less frequently. Further, it is increasingly recognized that individuals with SLSMDs may present with a range of manifestations that cross classical syndrome boundaries[4]. When SLSMDs are identified, they are typically thought to provide a definitive clinical diagnosis, with no need to pursue further diagnostic evaluation to discern their etiology.

**Fig 1:**
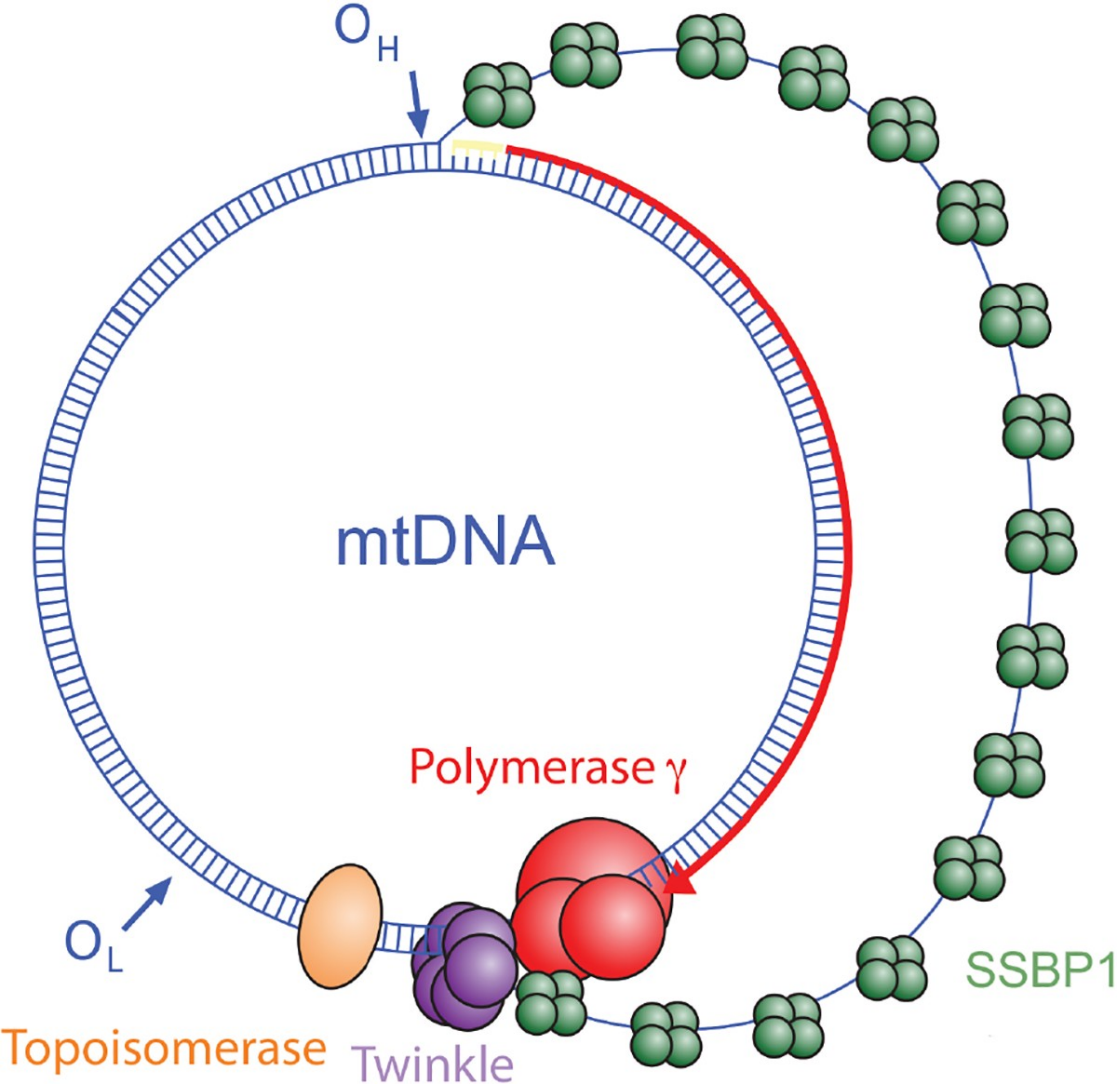
A schematic diagram of a mitochondrial DNA replication intermediate. Core replication proteins are illustrated: DNA polymerase γ (*red*), the Twinkle helicase (*purple*), mitochondrial single-stranded DNA binding protein, SSBP1 (*green*) and topoisomerase (*orange*). Image was adapted from [10] and updated based on recent findings [11].

mtDNA is replicated by a core collection of proteins that includes the DNA polymerase gamma (composed of the POLG catalytic subunit and POLG2 accessory subunit), the Twinkle helicase, and the single stranded DNA binding protein (SSBP1)[5–8] (Fig 1). Defects in *POLG*, *POLG2*, and *TWNK* are frequent causes of inherited forms of mitochondrial disease that include recessive and dominant forms of progressive external ophthalmoplegia (PEO), ataxia-neuropathy syndromes, childhood myocerebrohepatopathy spectrum disorders, and Alpers-Huttenlocher syndrome[9]. Multiple mtDNA deletions or mtDNA depletion are commonly identified in these nuclear gene disorders that impair mtDNA replication.

Single stranded DNA binding proteins (SSBs) bind and protect single stranded DNA (ssDNA) with very high selectivity and affinity during DNA replication[12]. Without SSB, ssDNA would be vulnerable to attack by chemical damage, nucleases, and DNA binding by inappropriate proteins[13, 14]. SSBs also help prevent secondary structure formation in ssDNA regions. In mitochondria, given the heavy concentration of 22 tRNA genes in mtDNA, secondary structure can represent a road-block during replication of the displaced single-stranded heavy strand initiated from the light strand origin of replication (OriL). Mitochondrial SSB was initially identified in *X*. *laevis* oocytes, with subsequent cloning of *Xenopus*, rat, and human SSB, all of which show high homology with *E*. *coli* SSB[15, 16]. Deletion of the yeast mitochondrial SSB (*RIM1*) causes loss of viability on non-fermentable carbon sources and loss of mtDNA[17]. Knock-down of SSBP1 expression in human cells causes loss of mtDNA and severely reduced synthesis of the 7S DNA[18]. A 2.4 Å resolution X-ray crystal structure of human mitochondrial SSBP1 in the absence of mtDNA reveals a tightly interacting tetramer formed by two dimers[19]. Although no structure of SSBP1 with mtDNA has been determined, we have observed ssDNA wrapping once around the tetramer by using atomic force microscopy[11]. Recently, two studies have reported pathogenic variants of SSBP1. The c.3G>A variant, which abolishes the SSBP1 start codon, co-segregates in one family with the m.1555A>G mtDNA variant underlying maternally inherited deafness [20]. Three missense variants, c.113G>A (p.R38Q), c.320G>A (p.R107Q), and c.422G>A (p.S141N), were reported in two families and two singletons with autosomal dominant optic atrophy and varying levels of retinal degeneration[21].

Here, we report a now 14-year-old Chinese boy who presented to the Children’s Hospital of Philadelphia (CHOP) Mitochondrial Medicine Frontier Program for clinical evaluation of lifelong, severe, progressive, multi-system disease. Indeed, he had progressive clinical manifestations across the full Pearson, Kearns-Sayre, and Leigh syndromes spectrum. mtDNA genome sequencing by next generation sequencing in his blood identified a SLSMD, a novel 5,440 base pair deletion (m.8629_14068del5440), which was subsequently determined by digital droplet PCR (ddPCR) to be present at 68% and 16% heteroplasmy in the proband’s fibroblast cell line and blood, respectively. Muscle biopsy was refused by the family due to risk of anesthesia. Given the complexity of his disease, whole exome trio sequencing in blood was performed to exclude an additional nuclear gene disorder, which demonstrated the proband had a novel *de novo* heterozygous variant c.79G>A (p.E27K) in *SSBP1*. Detailed *in silico* modeling and functional biochemical validation of p.E27K SSBP1 were performed to determine whether the mutation disrupts SSBP1 tetramerization, thermostability, molecular dynamics, and mtDNA binding capacity.

## Materials and methods

### Informed consent

The proband’s parents provided informed consent to participate in the Children’s Hospital of Philadelphia Institutional Review Board-approved protocol (#08–6177, M.J.F. PI).

### Mitochondrial DNA deletion detection in proband

Clinical diagnostic testing was performed to evaluate for mtDNA mutations or deletions. Specifically, whole mitochondrial genome sequencing and deletion (WMGSD) testing was performed in DNA extracted from early passage fibroblast cells and blood, and carried out by a commercial laboratory (GeneDx), following informed consent, according to previously described method[22, 23]. The identified large-scale mitochondrial deletion was confirmed by using junction PCR-Sanger sequencing and quantified by using a custom-designed oligonucleotide whole mitochondrial genome array-based comparative genomic hybridization (WMG-aCGH)[24].

Subsequently, research-based mtDNA genome sequencing was performed in the CHOP Division of Genomic Diagnostics to confirm and determine the degree of heteroplasmy of the mtDNA deletion. Specifically, mtDNA was enriched from early passage fibroblast cells and blood by amplifying the entire mitochondrial genome using custom primers and previously published primers[25] modified to work with Invitrogen Platinum SuperFi DNA Polymerase long-range PCR reagents (ThermoFisher). Libraries for each amplicon were prepared using a modified SureSelect QXT (Agilent) protocol and run on an Illumina HiSeq2500 to attain an average coverage of greater than 20,000X. Sequences were processed using a custom bioinformatics pipeline that utilized Novalign v3.06 for sequence alignment and FreeBayes v1.01 for variant calling, including heteroplasmy down to 1%. Haplogroups were determined using HaploGrep v2.1.1, and variants were annotated using data from SnpEff v4.3T, MitoMap v20180625 and Mamit-tRNA. Deletions were identified visually in Integrative Genomics Viewer (IGV). The deletion observed in this proband was quantified using droplet digital PCR by comparing the percentage of droplets from a region within the mitochondrial deletion to that of a non-deleted region of mtDNA. Two sets of probes were run in triplicate, where one assay targets a region that falls within the deletion area to represent non-deleted mtDNAs. The other probe targets an area outside of the deletion to represent the total mtDNA. The heteroplasmy level is calculated by taking 1 minus the ratio of non-deleted over total mtDNAs. The error is a 95% confidence interval assuming a normal distribution.

### Whole exome sequencing in proband and parents’ blood

Informed consent was obtained for whole exome sequencing (WES) together with repeat WMGSD testing on a clinical diagnostic basis by a commercial laboratory (GeneDx). WES was performed using a trio-based design, as previously described[22, 23]. Specifically, genomic DNA from whole blood was extracted and isolated from the affected proband and his parents using standard methods. Exome re-analysis was later performed when additional clinical information on the proband was received by the diagnostic laboratory, where previously generated whole exome sequence data from this individual's genomic DNA sample was re-annotated and re-analyzed in comparison with the published human genome build GRCh37/UCSC hg19 and analyzed for sequence variants using a custom-developed analysis tool (Xome Analyzer). The targeted coding exons and splice junctions of the known protein-coding RefSeq genes and the average depth of coverage and data quality threshold values remain the same from the initial analysis. Capillary sequencing or another appropriate method was used to confirm all potentially pathogenic variants identified in the proband. Sequence and copy number alterations were reported according to the Human Genome Variation Society (HGVS) and International System for Human Cytogenetic Nomenclature (ISCN) guidelines, respectively. Variants identified in WES were evaluated and classified according to the published American College of Medical Genetics and Genomics (ACMG) and the Association for Molecular Pathology (AMP) guidelines[26].

### Cell culture

Human fibroblast cell lines (FCLs) and human lymphoblastoid cell lines (LCLs) were established in the CHOP Division of Genomic Diagnostics. Coded cell lines were studied in accordance with the terms of the written informed consent. Briefly, FCLs were cultured in a mixture of DMEM (Gibco) containing 10% FBS (Gibco), 1 g/L glucose and supplemented with 1 mM sodium pyruvate (CellGro), 2 mM L-glutamine, and 50 μg/mL uridine (Calbiochem) since mitochondrial disease deficient lines become uridine auxotrophs[27]. LCLs were cultured in RPMI 1640 Medium supplemented with 10% fetal calf serum (FCS), 2 mmol/L L-glutamine, and 50 μg/mL uridine. Cells were incubated in a humidified environment at 37°C in 5% CO_2_[28].

### Mitochondrial DNA (mtDNA) copy number

Total DNA was extracted from human FCLs and LCLs using Qiagen DNA Mini extraction kit (Qiagen #51304) as per the manufacturer’s recommendations. DNA concentrations were measured using a Nanodrop 1000 spectrophotometer (Thermo Scientific). Genomic DNA stocks were subsequently diluted in water to a final concentration of 40 ng/ml. The cytochrome c oxidase subunit I (CO3) and NADH-ubiquinone oxidoreductase chain 1 (ND1) gene of the mtDNA and the B2M nDNA gene were amplified by qPCR (ABI 7500 Fast Real-Time PCR System). The primers are from ThermoFisher Scientific, including MT-CO3 (Hs02596866_g1); 4535. MT-ND1 (Hs02596873_s1); and β-2-microglobulin (β2M) (Hs06637353_s1). For PCR sample preparation, 5 ul of genomic DNA (40 ng/ml) was mixed with 1 ul of each primer (20x Taqman Gene Expression Assay), 4 ul of nuclease-free water, and 10 ul of Taqman gene Expression Master Mix. All the real-time PCR reactions were run in triplicate. Amplification curves were analyzed using SDS 1.9.1 software (Applied Biosystems), and these curves were used to determine the relative mtDNA: nDNA ratio in each sample.

### Western Blotting analysis

Fibroblasts from skin biopsy from the proband and father, and lymphoblasts from the proband and mother were harvested for immunoblot analysis by washing in phosphate-buffered saline (PBS) and lysing in RIPA lysis buffer (50 mM Tris, pH 8, 150 mM NaCl, 5 mM KCl, 5 mM MgCl2, 1% NP-40) supplemented with protease inhibitors (1:100, Sigma-Aldrich) and 0.5 mM PMSF. After incubation on ice for 20 min, samples were spun at 13,000 × g at 4°C for 5 min and supernatant was collected. Protein concentration was determined using DC protein Assay (Bio-Rad, Hercules, CA). Protein extract samples were run on 4–15% Tris-Glycine gradient gels (Bio-Rad). Separated proteins were transferred to nitrocellulose membranes (Bio-Rad, Hercules, CA), which were then blocked with Odyssey blocking buffer for 1 h and incubated with the primary antibodies (anti-SSBP1 rabbit polyclonal antibody, 1:750 dilution, Proteintech, #12212-1-AP), β-actin (1:2000, Cell Signaling) overnight and then Odyssey IRDye goat anti-rabbit or rabbit anti-mouse secondary antibodies at 1:10,000 for 30 min. Membranes were scanned directly using the Odyssey Infrared Imaging System (LI-COR Biosciences) and quantified with Image J software.

### Mitochondrial high-resolution respiratory capacity quantitation in intact cells by Oxygraph-2 K (Oroboros) analysis

For determination of respiratory activity in intact cells, cells were evaluated by high-resolution respirometry using the Oxygraph-2k (Oroboros Instruments, Austria). As previously described, two cell lines were simultaneously analyzed in two separate chambers in 2 mL volume containing 1 × 106 cells for FCLs and 4× 106 cells for LCLs per chamber. Respiration was measured in intact cells at 37°C in 2 mL culture medium (DMEM) for FCLs and RPMI1640 medium for LCLs and inhibitors for the different mitochondrial respiratory chain complexes were added into each chamber. Routine consumption, oligomycin-independent respiration (proton leak), FCCP-stimulated respiration (ETS capacity-maximum respiration) and ROX (residual oxygen consumption in the presence of rotenone and anti-mycin A) were measured in intact cells, as described previously[27]. After observing steady-state respiratory flux, the ATP synthase (complex V) inhibitor oligomycin (1 μg/mL, Sigma) was added, followed by uncoupling of oxidative phosphorylation by stepwise titration of FCCP (in 1.5 μMol increments, Sigma) to assess complex I and complex Il-uncoupled respiration. Finally, respiration was inhibited by the complex I and complex III inhibitors rotenone (0.5 μM, Sigma) and antimycin A (2.5 μM, Sigma), respectively. All values were corrected for ROX. DatLab software (Oroboros Instruments) was used for data acquisition and analysis. Statistical analyses were performed using ANOVA and paired t-tests to compare group means.

### Plasmids and oligonucleotide substrates

Site-directed mutagenesis was used to introduce c.79G>A into pET21aHmtSSB[29] to generate pET21aHmtSSB_E27K for expression and purification of the mutant protein. For both WT and p.E27K SSBP1, the cDNA is truncated to express only the mature protein, lacking the mitochondrial targeting sequence (amino acid residues 17–148). Synthetic fluorescein-labeled 50 nucleotide oligomer (5’- AAT GCT ATC ACT ATT CGT AGA CTT GAC CAC ACC TTG TCA GCT CAC GCT CC-FAM -3’) utilized to assess DNA binding affinity was purchased from Integrated DNA Technologies, resuspended in water, and quantified by absorbance at 260 nm.

### Purification of wild-type and E27K human SSBP1 protein

Recombinant wild-type (WT) and p.E27K SSBP1 were purified to homogeneity as previously[29] described with modifications detailed below. Plasmids were transformed into BL21(DE3) competent cells (Agilent), which were expanded in Luria Bertani broth and harvested by centrifugation. Following lysis by sonication in Lysis Buffer (30 mM HEPES-KOH, 50 mM KCl, 0.25 mM EDTA, 0.25% myo-inositol, 0.1 mM phenylmethylsulfonyl fluoride, and 1 mM dithiothreitol), clarified lysate was purified over Affi-Gel Blue resin as previously described. Affi-Gel Blue eluate was exhaustively dialyzed into Dialysis Buffer (50 mM CHES-KOH pH 9.5, 100 mM KCl, 0.1 mM EDTA, 10% glycerol, and 2 mM β-mercaptoethanol) before clarification and application to a MonoQ 5/50 GL column (GE Healthcare) as previously described. MonoQ fractions containing protein of interest were pooled, mechanically concentrated, and applied to a Superdex 200 10/300 GL column (GE Healthcare) using an AKTA FPLC system (GE Healthcare) in Storage Buffer (30 mM HEPES-KOH pH 7.6, 250 mM KCl, 0.25 mM EDTA, 2 mM dithiothreitol, and 10% glycerol). Fractions containing protein of interest were pooled and mechanically concentrated. Final protein concentration was determined relative to known standards by densitometry following SDS PAGE. Protein was flash frozen and stored at -80°C.

### Differential scanning fluorimetry

WT and p.E27K SSBP1 at a final concentration of 1.5 mg/ml and SYPRO Orange (Molecular Probes) at a final ratio of 5X were combined in DSF Buffer (30 mM HEPES-KOH pH 7.6, 50 mM KCl, 0.25 mM EDTA, 2 mM dithiothreitol, and 10% glycerol), before transferring 20 ul of each sample to a 96-well plate in quadruplicate to account for well-to-well variability. Using a QuantStudio 7 Flex Real-Time PCR System (Applied Biosystems), samples were heated from 25°C to 95°C at a ramp rate of 1.6°C/s with detection set to the ROX reporter and with the x1-m3 filter in place. Data were collected using QuantStudio 7 Flex software (Applied Biosystems) and analyzed using Protein Thermal Shift v1.3 (Applied Biosystems). The peak of the first derivative of the thermal melting curve defines the melting temperature (T_m_) for each protein. Melting temperatures were averaged across plate quadruplicates and then triplicate experiments before statistical analysis by an unpaired t test with a Welch’s correction using GraphPad Prism software.

### DNA binding assessed by fluorescence anisotropy

Steady state fluorescence anisotropy was measured as previously reported[11], with an OLIS RSM1000 spectrofluorometer (Bogart, GA) equipped with a 1.24-mm slit and a temperature-controlled cell set to 37°C. Incident light at a 480 nm excitation wavelength was horizontally plane-polarized and passed through a T-format quartz fluorometer cell, and a photoelectric modulator was utilized to simultaneously measure horizontally and vertically plane-polarized fluorescence at 530 nm with a gated photon counting detector. Binding mixtures (1 ml) contained 30 mM HEPES-KOH pH 7.6, 50 mM KCl, 2 mM dithiothreitol, 5 mM MgCl_2_, and 20 nM fluorescein-conjugated oligonucleotide substrate. Changes in fluorescence polarization were measured in response to the step-wise, small volume addition of purified WT or p.E27K SSBP1 in storage buffer. Following a one-minute equilibration period after each addition, anisotropy data were collected in triplicate with a 4 second integration time. Changes in anisotropy were plotted against the total concentration of SSB, expressed as tetramers. To correct for the ligand depletion effect caused by non-trivial concentrations of protein-DNA complex relative to the total protein concentration, binding isotherms were fit to a quadratic equation by non-linear regression analysis to calculate apparent *K*_*d*_(DNA) values[30] for triplicate experiments using GraphPad Prism software. Intrinsic fluorescence of buffer components was undetectable at wavelengths relevant to fluorescein.

### Molecular dynamics simulations

Using molecular dynamics, solution structures of SSBP1 WT and p.E27K (in monomer, dimer, and tetramer conformations) were generated. The initial structure of SSBP1 for simulations was taken from the homodimeric X-ray crystal structure from the PDB code 3ULL[19]. Positions of the peptide segments of 1–25 (N-terminus), 68–76, and 141–148 (C-terminus) from chain A and 1–25, 68–80, and 142–146 from chain B were not reported in the X-ray crystal structure due to poor electron densities. Using Modeller 9.17[31] missing residues were introduced and the reconstructed peptide segment 26–140 was used in simulations. The initial tetramer structure was created after aligning two dimer units to the tetramer in the structure of *E*. *coli* SSB (PDB ID: 1EYG). The E27K mutation was introduced using the program Coot-0.8[32] and 5 heterogeneous tetramer systems with a single mutant monomer, two mutant monomers (with two different monomer selections), three mutant monomers, and four mutant monomers were constructed. After introducing the protons using Molprobity[33], counter ions were added, each system was solvated in a box of water, and each solvent box was selected so that box boundaries were at least 20 Å from the closest protein atom (total atoms in each system ranging from 184,400 to 184,416). Prior to equilibration, all systems were subjected to 1) 100-ps belly dynamics runs with fixed peptide, 2) minimization, 3) low temperature constant pressure dynamics with fixed protein to assure a reasonable starting density, 4) minimization, 5) step-wise slow heating molecular dynamics at constant volume, and 6) constant volume unconstrained molecular dynamics for 20 ns. All final unconstrained trajectories were calculated at 310 K under constant pressure (for 300 ns with time step 1 fs) using the PMEMD module of Amber.18[34] to accommodate long range interactions. All protein parameters were taken from the FF14SB force field. Free energies of interactions were calculated using the MMBPSA module of Amber.18.

## Results

### *SSBP1 de novo* dominant mutation identified in proband with SLSMD and complex mitochondrial disease phenotype

**Fig 2:**
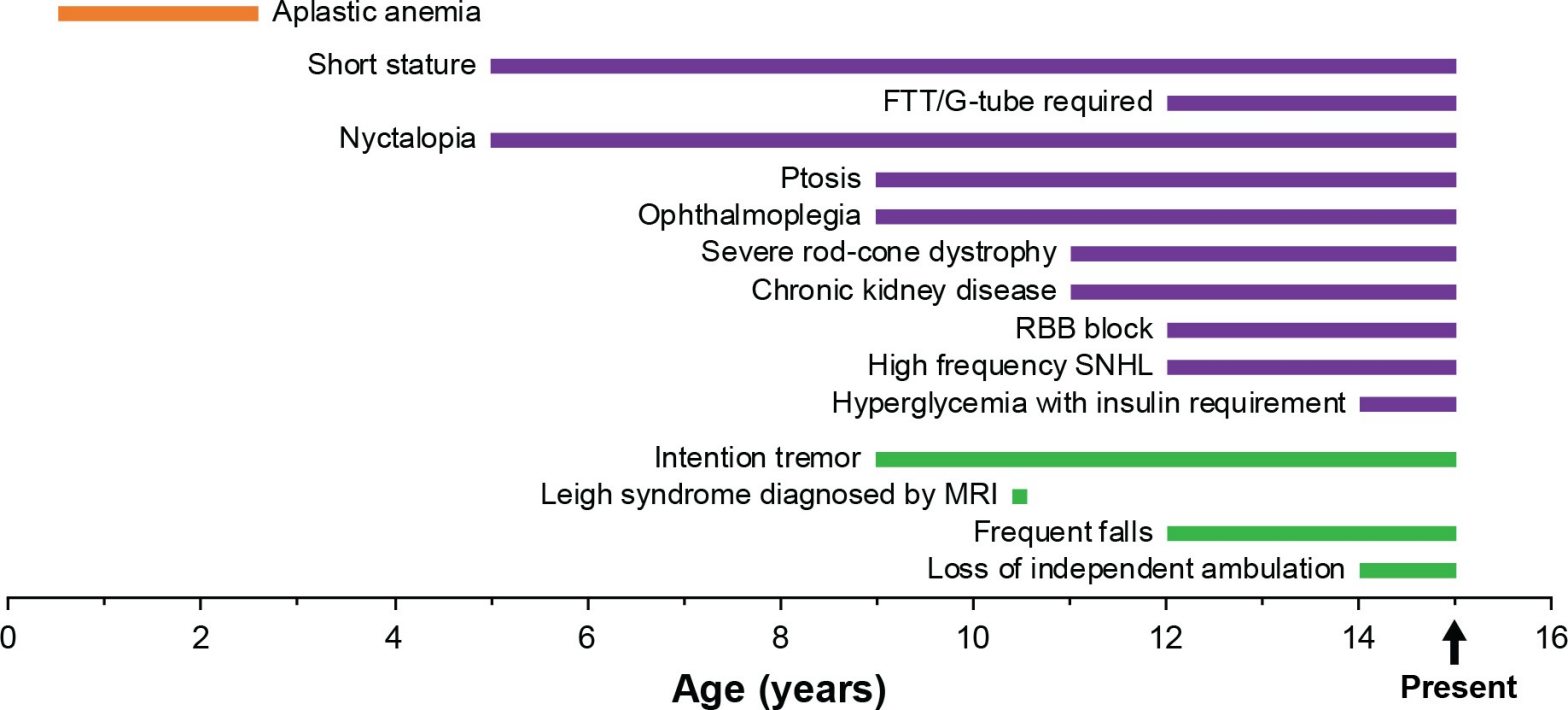
Timeline of proband’s mitochondrial disease clinical progression. Clinical manifestations associated with Pearson (orange), Kearns-Sayre (purple), and Leigh (green) syndromes are grouped. Manifestations through 14 years are indicated as ongoing to present.

A Chinese boy, presently 14 years old, was referred to the CHOP Mitochondrial Medicine Frontier Program for diagnostic evaluation at 11 years of age for progressive multi-system involvement suspected to be primary mitochondrial disease. While the mother had a normal pregnancy and the child showed normal early development except for delayed speech, he developed infantile-onset transfusion-dependent anemia and bone marrow failure, growth failure with frank growth hormone deficiency, and failure to thrive. He later developed progressive ataxia, ptosis, and ophthalmoplegia, followed by severe rod-cone dystrophy, sensorineural hearing loss, chronic kidney disease, cardiac conduction block, multiple endocrine deficiencies, and progressive neurologic decline including metabolic strokes consistent with Leigh syndrome (Fig 2). Laboratory testing revealed lactic acidemia (2.4–4.7 mM, normal <2 mM) as well as elevated CSF lactate (>2-fold increase) and CSF protein (>4-fold increase). Muscle biopsy was refused due to anesthesia concerns. Full clinical details are included in the supplement including full clinical history and laboratory testing results (S1 File), brain MRI images (S1 Fig), fundus photographs and optical coherence tomography (OCT) image (S2 Fig), audiogram (S3 Fig), and growth parameters (S4 Fig).

**Fig 3:**
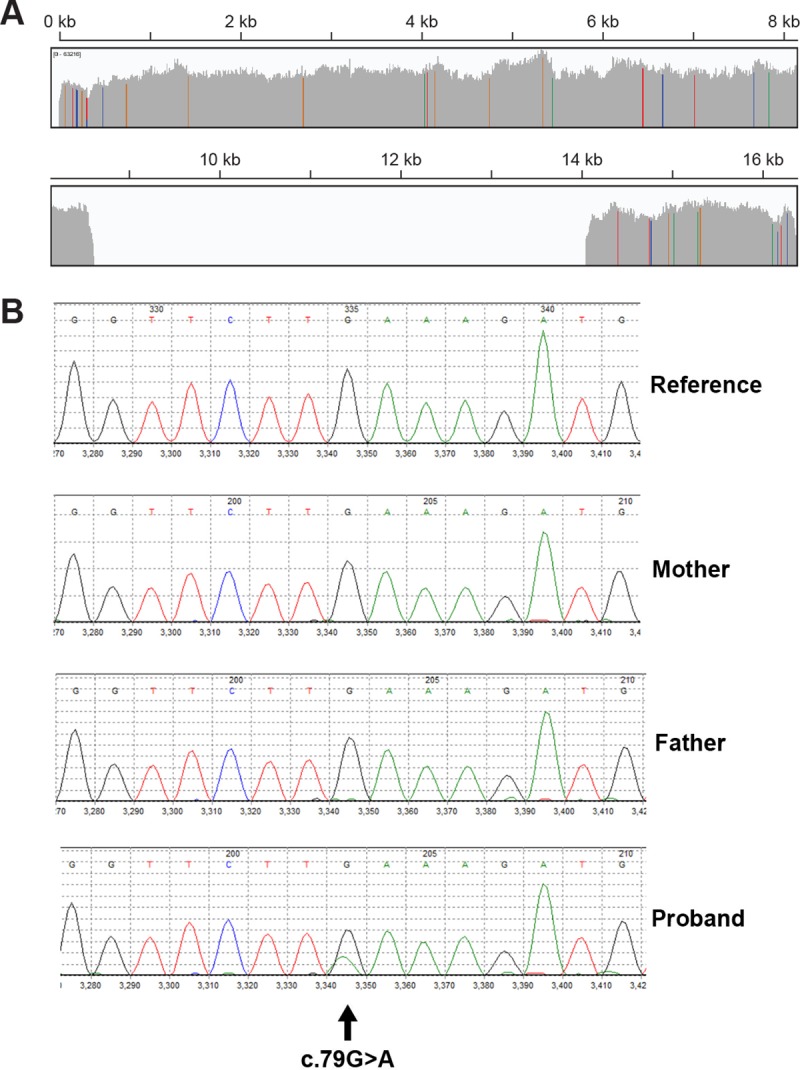
Mitochondrial disease proband with complex disease manifestations was discovered to harbor a SLSMD and a *de nov*o dominant *SSBP1* novel variant of uncertain significance. **A.** mtDNA deletion (m.8629_14068del5440) was detected in proband blood by NGS. The vertical gray bars represent absolute coverage of next generation sequencing reads for each base position of the reference mitochondrial genome. Green, blue, red and orange vertical bars indicate base pair differences from the reference (rCRS). The drop in coverage represents the large scale mitochondrial DNA deletion. Base positions are represented across the top of the figure. The depth of coverage shown in this figure is not reflective of total percent of deleted DNA in the starting population. **B.** *SSBP1 de novo* c.79G>A variant was identified by exome sequencing.

mtDNA genome sequencing by next generation sequencing in his blood on a clinical diagnostic basis at GeneDx identified a unique SLSMD, a 5,440 base pair deletion (m.8629_14068del5440) (Fig 3A). This mtDNA deletion was further confirmed by independent NGS analysis of the mtDNA genome at the CHOP Division of Genomic Diagnostics, with ddPCR analysis demonstrating this SLSMD was present at 68±3% and 16±5% heteroplasmy in the proband’s fibroblast cell line and blood, respectively. Given the severity and complexity of his disease manifestations, whole exome trio sequencing was performed to exclude additional nuclear gene disorders. mtDNA genome sequencing repeated by GeneDx at the time of exome sequencing again confirmed the presence in the proband of the same 5,440 base pair deletion (m.8629_14068del5440). Trio exome sequencing revealed the proband harbored a novel *de novo* heterozygous mutation c.79G>A in *SSBP1* that was absent in both parents (Fig 3B). This mutation was not present in dbSNP (https://www.ncbi.nlm.nih.gov/snp), MSeqDR (https://mseqdr.org/), nor ClinVar (https://www.ncbi.nlm.nih.gov/clinvar/) databases for the human SSBP1 and results in a glutamate (E) to lysine (K) substitution at residue 27 of mitochondrial single stranded DNA binding protein (SSBP1). This mutation was also not found in over 4,500 healthy Chinese individuals from the Chinese Millionome Database (CMDB) at China National GeneBank[35], nor another 5,333 East Asians by WGS including the 3.5KJPNv2 whole-genome reference panel[36] from 3,552 healthy Japanese individuals and the Northeast Asian Reference Database (NARD) derived from WGS data of 1,781 individuals from Koreans, Mongolians and other East Asians [37]. Furthermore, no mutations were found in known nuclear genes involved in mtDNA maintenance including, but not limited to, *POLG*, *POLG2*, *TWNK*, *RRM2B*, *DGUOK*, *TK2*, *SLC25A4*, *MPV17*, *MGME1*, and others.

While a muscle biopsy was not available for mtDNA copy number analysis, primary fibroblast cell line and lymphoblastoid cell lines from the proband and parents (as available) were analyzed for SSBP1 protein, oxidative phosphorylation capacity, and mtDNA copy number. As anticipated, no difference was observed in the level of SSBP1 protein between proband and father fibroblasts by Western blot analysis (S5A Fig). Mitochondrial respiratory capacity as measured by Oxygraph 2K (Oroboros) showed a 20% decrease in basal and maximal respiratory capacity of the proband as compared to fibroblasts from the father, and a 17% decrease in lymphoblastoid cells compared to the mother (S5B Fig). Coincident with this drop in mitochondrial respiratory capacity, a 50% decreased mtDNA copy number was observed in lymphoblastoid cells of the proband as compared to the mother using qPCR against *mt-ND1* (not affected by the deletion) and *mt-COXIII* (affected by the deletion). In contrast to the lymphoblastoid cells, an increase was found in fibroblasts as compared to the father, probably as a compensatory increase (S5C Fig).

### SSBP1 E27 is conserved among higher eukaryotes and may participate in an inter-subunit salt bridge

**Fig 4:**
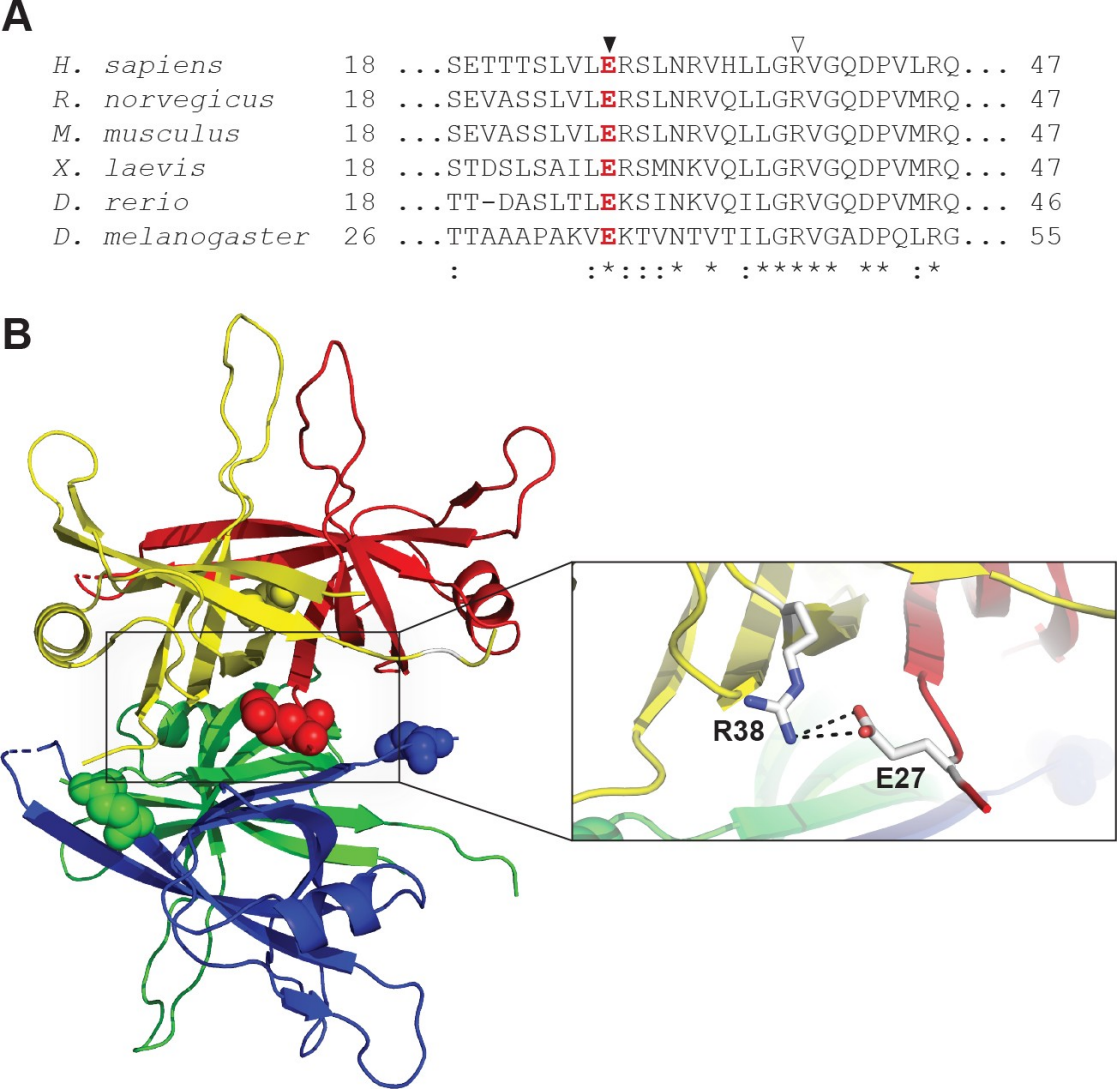
Glutamate 27 of SSBP1 is conserved among higher eukaryotes and may participate in a salt bridge at dimer interfaces. **A.** Amino acid sequence alignment of SSBP1 from six species shows that E27 (▼) is conserved among higher eukaryotes (*, invariant conserved amino acids; :, highly conserved sites). **B.** E27 residues are highlighted as ball models on a ribbon diagram of the structure of the SSBP1 tetramer (PDB 3ULL). The inset shows that E27 in each monomer may participate in a salt bridge with R38 (▽ in **A**) from its respective adjacent monomer. The distances shown for the interactions in this potential salt bridge are 3.4 Å (top) and 3.1 Å (bottom).

Multiple sequence alignment of the primary sequences of mitochondrial SSBs from several species reveals that E27 is invariant among higher eukaryotes (Fig 4A). SSBP1 forms a homotetramer, and E27 is found at monomer-monomer interfaces when mapped onto a tetramer modeled from the reported crystal structure of SSBP1 (PDB 3ULL[19]) (Fig 4B). E27 resides at the edge of a positively-charged surface patch which may be involved in DNA binding. The patch also harbors two arginine (R) residues implicated in mitochondrial disease: heritable pathogenic missense variants of R38 or R107 to glutamine (Q) were recently reported[21]. Importantly, E27 appears well-positioned to participate in an inter-subunit salt bridge with R38 in the neighboring subunit at each monomer-monomer interface (Fig 4B, inset), an interaction which may be structurally significant for the tetramer.

Several testable hypotheses for a molecular mechanism of disease for E27K present themselves. First, the charge inversion from glutamate to lysine may break the putative inter-subunit salt bridge and destabilize the tetramer. Second, the loss of negatively-charged glutamate and addition of positively-charged lysine could strengthen DNA binding through the net increase of positive charge. Third, the replacement of glutamate with lysine might rearrange the adjacent positively-charged patch, altering DNA binding or protein stability. To investigate the biochemical consequences of the E27K charge inversion, we purified recombinant WT and p.E27K SSBP1 overproduced in *E*. *coli*.

### p.E27K SSBP1 remains a stable tetramer but is less thermostable than WT SSBP1

**Fig 5:**
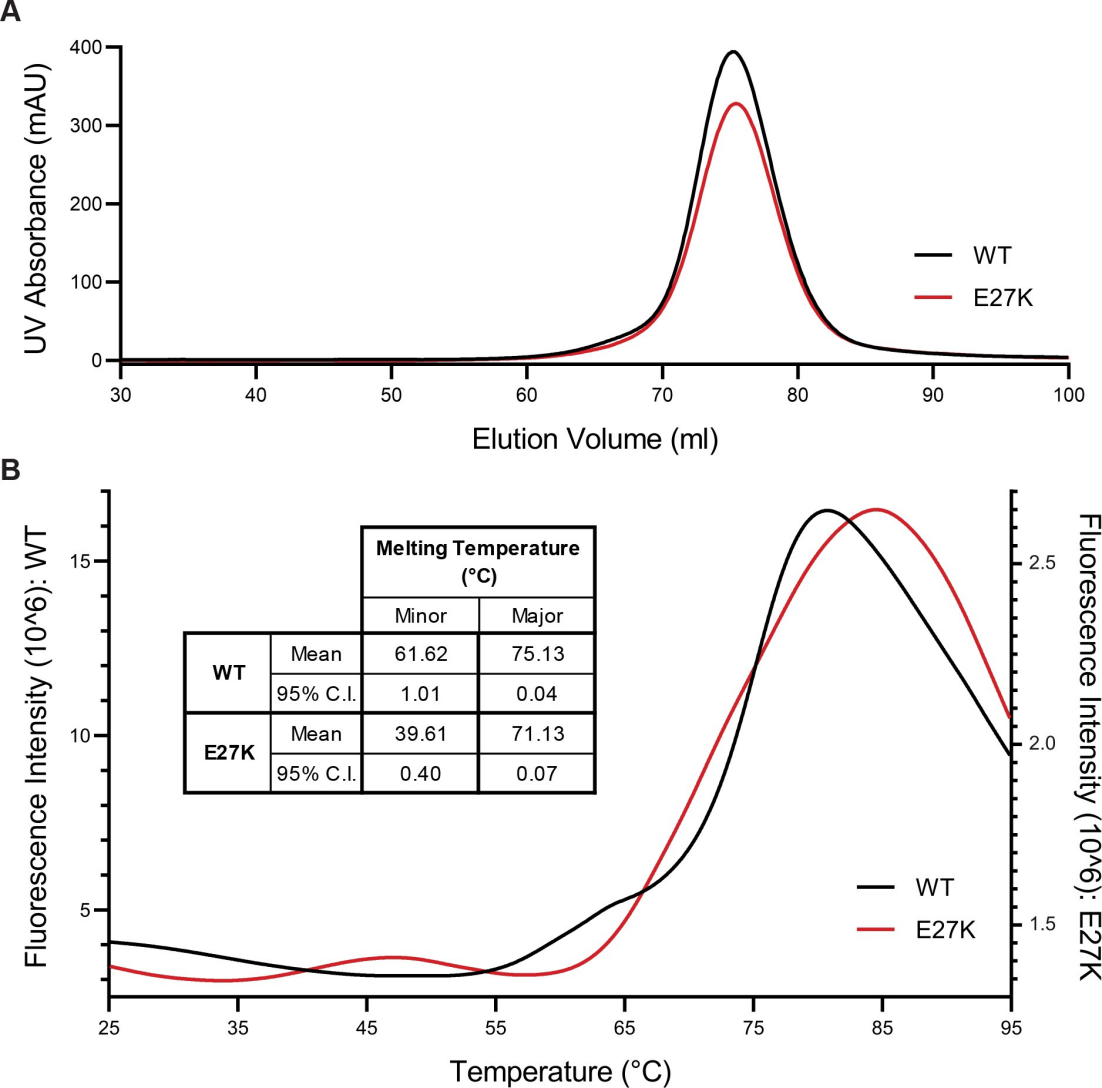
Purified p.E27K SSBP1 is a stable tetramer but is slightly less thermostable than WT SSBP1. **A.** UV intensity measurements are plotted against elution volume for size exclusion chromatography of WT SSBP1 and p.E27K SSBP1. **B.** Representative thermal denaturation curves for WT SSBP1 and p.E27K SSBP1 are overlaid. Inset table lists average melting temperatures for minor (leftmost) and major (rightmost) melting peaks for each protein (n = 3). Differences between values for WT and p.E27K at minor and major melting peaks are statistically significant (minor, p = 0.0001; major, p < 0.0001).

The simplest hypothesis for a molecular mechanism for disease is that the p.E27K variant breaks the SSBP1 tetramer. If this were the case, size exclusion chromatography would reveal multiple species of different sizes, representing tetramers, dimers, and monomers. However, size exclusion chromatography of WT SSBP1 showed a single symmetrical peak, consistent with the documented stability[38] of SSBP1 as a tetramer (Fig 5A). p.E27K SSBP1 also eluted from this column in a single peak at the same elution volume, indicating that the E27K substitution does not destabilize the tetramer under these conditions (Fig 5A).

Even with a stable tetramer, changes in the overall stability of p.E27K SSBP1 may point to a mechanism for disease. To test whether the overall thermostability of SSBP1, as a property of the protein, might be affected by the E27K substitution, both WT and p.E27K SSBP1 were subjected to differential scanning fluorimetry (Fig 5B). WT SSBP1 yielded a major melting temperature of 75.1°C, consistent with previous reports, while p.E27K SSBP1’s major melting temperature was shifted lower, to 71.1°C (Fig 5B, inset). Both proteins also showed a minor melting peak, observed for WT SSBP1 as a shoulder with a melting temperature of 61.6°C and for p.E27K SSBP1 as a small distinct peak with a melting temperature of 39.6°C (Fig 5B). These complex melt curves are unsurprising for tetrameric complexes, which pass through multiple stages during thermal denaturation. Further, both proteins are very stable at physiologically relevant temperatures. Intriguingly, the signal intensity for p.E27K SSBP1 was reproducibly low compared to that of WT SSBP1, regardless of salt or protein concentration, suggesting some fundamental difference in binding of the fluorescent dye employed in the assay. Overall, these data indicate small changes exist in the thermostability of p.E27K SSBP1 relative to WT SSBP1.

### E27K does not substantially weaken SSBP1 DNA binding, but may alter DNA binding behavior

In the absence of a gross destabilizing effect of the p.E27K SSBP1, we next interrogated the biological function of SSBP1, ssDNA binding. Of the available methods for assessing DNA binding, fluorescence anisotropy has the advantage of permitting the observation of protein-DNA interactions in solution. This eliminates the potential for effects of the molecular environment encountered by the proteins and DNA during electrophoresis. Fluorescence anisotropy reflects the mobility of a fluorophore attached to the DNA substrate. An increase in anisotropy signals decreased mobility of the DNA substrate, indicative of a protein-bound state. Therefore, anisotropy should increase with protein concentration, reaching a final plateau at saturation of DNA-protein binding. An apparent *K*_d_ can be calculated from a quadratic fit of these data points. In the case of p.E27K SSBP1, three results were possible relative to WT: tighter binding (lower *K*_d_), weaker binding (higher *K*_d_), or no change in binding of ssDNA (the same *K*_d_).

**Fig 6:**
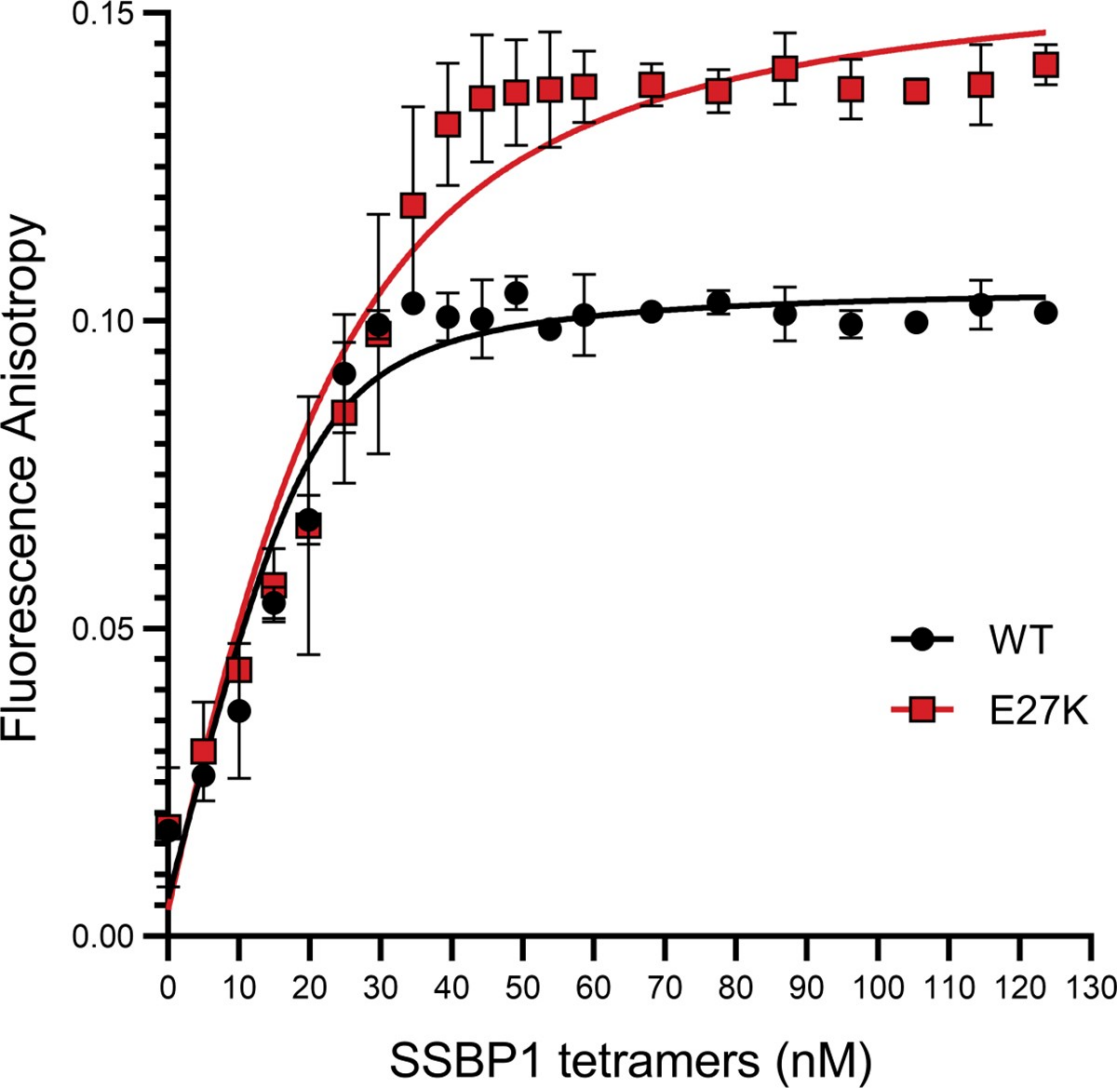
p.E27K SSBP1 binds ssDNA subtly differently than WT SSBP1. Changes in fluorescence anisotropy of 20 nM fluorescein-labeled 50 nucleotide ssDNA substrate were measured in response to the step-wise addition of WT or p.E27K SSBP1. Nonlinear regression fits are plotted as thick lines for each dataset. Protein concentration refers to tetramers. Error bars represent 95% confidence intervals; n = 3.

When we measured fluorescence anisotropy of a FAM-labeled 50 nucleotide ssDNA substrate in the presence of increasing concentrations of SSBP1 tetramer, we determined an apparent *K*_d_ for WT SSBP1 of 2.3 nM (S.D. ± 0.82) (Fig 6). For p.E27K SSBP1, we determined an apparent *K*_d_ of 9.3 nM (S.D. ± 1.32). While there is a 4-fold difference between these apparent *K*_d_ values, they both reflect very tight binding and are consistent with no major defect in the affinity of p.E27K SSBP1 for ssDNA.

Intriguingly, the maximum anisotropy values for p.E27K SSBP1 at its plateau are about 1.4-fold higher than those for WT. This indicates that at saturation, where every ssDNA is bound to SSBP1, the fluorophore on the ssDNA bound to p.E27K SSBP1 is less mobile than that associated with WT SSBP1. Control experiments confirmed that our 50 nucleotide ssDNA substrate binds only one tetramer of either WT or p.E27K SSBP1 (S6 Fig). Therefore, the simplest explanation for this observation is that the ssDNA end to which the fluorophore is attached may somehow be more tightly “tucked” against the protein in the case of p.E27K SSBP1 than in WT. In the absence of a crystal structure with DNA bound, any proposal about the nature of this “tucking” is purely speculative.

Based on the collective biochemical data, no clear molecular mechanism for disease presents itself. With only modest differences in thermostability and DNA binding between WT and p.E27K SSBP1, it seems likely that a more elusive biological explanation exists. One tantalizing possibility is that the *in vivo* defect arises from the fact that the proband is heterozygous for the disease allele. This means that SSBP1 tetramers within the proband’s mitochondria would be a mixture of WT and p.E27K homotetramers, as well as an array of heterotetramers in various combinations. The biochemical studies presented have focused on the homotetramers, as the heterotetramers are biochemically impossible to reproduce. SSBP1 monomers are bound so tightly within each tetramer as to preclude disassembly of homotetramers and reassembly into heterotetramers *in vitro*. And while intricate purification strategies might make purification of heterotetramers possible, these heterotetramers would exist in mixtures of orientations of WT and p.E27K monomers within each tetramer, rendering experimental interpretation impossible. To circumvent this experimental obstacle, we turned to molecular modeling as a method for ascertaining differences between WT SSBP1 and variations of the tetramer with p.E27K SSBP1.

### *In silico* modeling of the WT and E27K SSBP1 confirms the stability of tetramers with various mutant monomer compositions

Molecular dynamics (MD) simulations have proven to be a useful tool in unearthing structure-function relationships in recent years, specifically in cases where mutations may alter structural and thermodynamic properties of proteins. In the present case, we can reliably use the existing structural information of mitochondrial SSBP1 available from the Protein Data Bank to predict solution structures of WT SSBP1 and to construct mutant solution structures to study changes in mutant proteins. Several solution structures were estimated for various selected systems including the WT tetramer (WT-abcd, where “a, b, c, and d” are the monomer identities used in the text hereafter), and five tetramer conformations with the E27K mutation introduced in only monomer a, monomers a and b, monomers a and c, monomers a, b, and c, and all monomers.

First, we confirmed the stability of mutant tetramer structures through the root mean square deviations (RMSD) from our MD trajectories via the observation of the convergence of RMSD values during the last 100 ns of simulations (S7 Fig). We have noted that the two monomers reported in the X-ray crystal structure of human SSBP1 showed a RMSD value of 1.8 Å for their backbone conformations, and a segment of residues in the middle of each peptide was found to be disordered[19]. All of our solution structures converged to yield RMSDs in the range of 3–4.5Å. This convergence indicates stable tetramers for WT and p.E27K SSBP1 in solution, consistent with our experimental size exclusion results. Observed RMSD values for structures of proteins of this size are generally smaller than those for both the crystal and solution structures of SSBP1. This suggests that SSBP1 is more dynamic in solution than other proteins of comparable size. This dynamic nature may potentially be required by the function of SSBP1 since ssDNA needs to be loaded preferentially and with high affinity.

### Molecular dynamics simulations reveal constrained mobility in p.E27K SSBP1 tetramer

**Fig 7:**
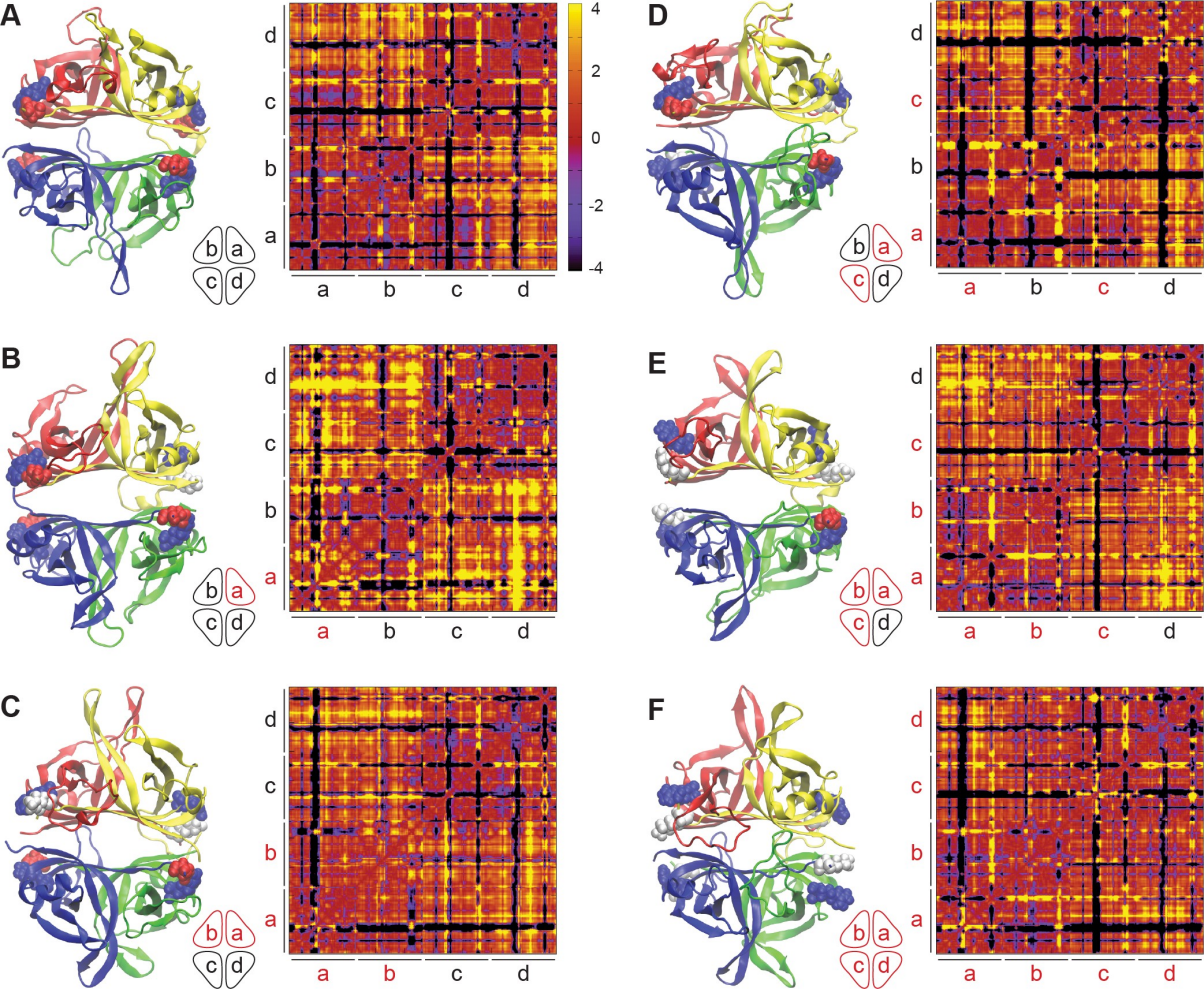
Tetramer stability is illustrated by snapshots of the representative conformations obtained from the latter part of each molecular dynamics trajectory. The six configurations simulated are shown (**A-F**). Each monomer is color-coded (a, yellow; b, red; c, blue; d, green) and E27 is shown in red solid spheres with its salt bridge residue R38 (in blue). The mutant residues (K27) are shown in white spheres. Heat maps of deviations of C_α_ distances calculated from the last 100 ns of each trajectory are shown next to each structure. Mutant monomers are indicated in red in the cartoons and the axis labels. Large negative and positive C_α_ deviations are displayed in black (closer in space) and yellow (farther in space), respectively.

Representative structures created using snapshots taken from the latter part of the MD trajectory of each system are shown in Fig 7. In all systems studied, the inner β-barrel_BARREL_ scaffold is preserved and, as it does in the WT, seems to provide the necessary stability at the tetramer interface (Fig 7). The inter-monomer interaction free energy (S1 Table) predicts possible variations in the strength of inter-monomer interactions. Large shifts in free energy among various monomers are observed when some or all monomers contained mutated E27K, and certain combinations show strengthened interactions between some monomers without weakening of other interactions. This may be viewed as a gain of strength in tetramer formation in the presence of one or more mutant monomers.

In contrast to this observation, mobility for all models is much more apparent in outer loops that may be in contact with ssDNA. This mobility can be visualized with heat maps representing the averaged deviations in alpha carbon (C_α_) distances (Fig 7). Such C_α_ deviation maps convey the positional fluctuations of each amino acid residue at its base with respect to any other residue’s C_α_, producing a summary of amino acid residue movements within a protein. In this case, C_α_ deviations were evaluated by averaging C_α_ distances of the tetramer configurations from the final 100 ns of each simulation and subtracting the initial C_α_ distances, to reflect only changes in C_α_ distances over the simulation. All systems exhibit combinations of stable (red) and dynamic (yellow or black) peptide regions; in short, these maps indicate a high degree of mobility. Regions with smaller deviations (red) are mostly the residues providing the stability in the fold of each monomer and in tetramer interactions. Solvent-exposed residues, loops, and β-hairpins in each monomer experiences larger deviations (yellow or black). This is further supported by the root mean square fluctuations of each residue calculated through normalized B-factors (S8 Fig). Large fluctuations are observed in the N-terminus and in all three β-hairpin loop regions (bracketed by residues 46–53, 67–81, 119–126). These data suggest that several regions have relatively large fluctuations, irrespective of the tetramer system, and individual monomers show variable degrees of fluctuation when homologous segments are compared. This also indicates that the symmetry shown in the X-ray crystal structures doesn’t hold for peptides in the outer loops.

To further quantify these data, we calculated the percentage of residue pairs fluctuating within a given distance as a semi-quantitative measure of the mobility of each tetrameter system. Intra-monomer fluctuations reveal an important difference between residue mobility in WT and p.E27K monomers. In WT monomers, 71% of intra-monomer deviations are within 2 Å or less for each monomer in the tetramer. Interestingly, this number increases to 77% for monomers in the averaged mutant systems. The fact that only 23% of residue pairs in the mutant systems have larger than 2 Å deviations, as opposed to 29% of pairs in the WT, supports the interpretation that mobility of the mutant tetramers is constrained. When overall (intra- and inter-monomer) fluctuations are taken into consideration, similar differences are observed for WT (61%) and the mutants (69%), representing a 7% decrease in the deviations larger than 2 Å in the mutant systems.

### Net positive charges reflected in solvent accessible surface areas are significantly different in WT and p.E27K tetramer solution structures

Our estimated solution structures also allow us to address the effect of the additional charge that is introduced by the E to K substitution. Whenever an E residue is mutated to a K, each monomer peptide increases its net electronic charge by +2e. This introduces +2e, +4e, +6e, and +8e additional charges on tetramer assemblies when one, two, three, and all four monomers are mutated, respectively. In the WT SSBP1 tetramer, there exists a net +16e charge due to the counter balancing of 68 positively-charged K and R residues by 52 negatively charged E and aspartate (D) residues. To study how this net positive charge is distributed on the molecular surface, we constructed electrostatic potential (ESP) maps for each system using the partial atomic charges available from the same force field used in MD trajectory calculations. Such ESPs serve as reasonable estimates for the interacting surface presented to negatively charged ssDNA. The ESP surfaces calculated on representative conformations are displayed in S9 Fig, and positively-charged (blue) patches that are potentially used in recruiting ssDNA segments can be readily observed on these surfaces. Additionally, a quantification of the charge distribution in a global sense is presented in Table 1. A slight reduction in total surface area is observed for the mutant systems. Unexpectedly, a reduction in the positively-charged area relative to WT is observed for tetramer systems with two or fewer mutant monomers, while this area is increased in the other two systems (tetramers with three or four mutant monomers). A significant reduction in overall positively-charged surface area is observed for the system with a single monomer mutant. In contrast, a substantial increase in the area with a net positive surface charge is observed when three or more monomers in the tetramer are mutants. Such variations in the area with a net positive charge might have a significant effect on DNA binding *in vivo*, where a mixture of tetramers would be available to bind mtDNA and variability from tetramer to tetramer could impact overall protection of ssDNA during replication.

**Table 1:** Averaged total solvent accessible areas (SAAs) of WT and p.E27K SSBP1 were calculated from the last 100 ns of the molecular dynamics trajectory. The SAAs of positively-charged residues (R and K) and negatively-charged residues (E and D) are also shown, along with the net positively-charged SAA calculated from the difference between positive and negative SAA. Monomers underlined are E27K. Highlight colors reflect differences from the WT tetramer in each category: yellow indicates reduction; blue indicates increase. ± reflects standard deviations.

	Solvent Accessible Area (Å^2^)			
	Total	Positive (Lys, Arg)	Negative (Glu, Asp)	Net Positive
**abcd**	28158 ± 286	7125 ± 122	3724 ± 102	3401 ± 112
**a****bcd**	26379 ± 262	6337 ± 147	3742 ± 93	2595 ± 117
**ab****cd**	27092 ± 320	6744 ± 120	3418 ± 99	3326 ± 109
**a****b****c****d**	27142 ± 289	6783 ± 132	3503 ± 80	3220 ± 103
**abc****d**	28085 ± 206	7296 ± 123	3565 ± 91	3731 ± 106
**abcd**	27712 ± 369	7386 ± 134	3475 ± 125	3911 ± 129

## Discussion

We present here the first report of a nuclear gene defect associated with SLSMD in a 14-year-old Chinese proband with severe, progressive, multi-system manifestations of all classical SLSMD clinical syndromes, including Pearson, KSS, and Leigh syndromes. Specifically, we identified and functionally validated that a novel *de novo* dominant missense variant c.79G>A in the nuclear gene *SSBP1* leads to biophysical changes in its protein product, p.E27K SSBP1. Our analyses suggest several mechanisms by which various tetramer combinations harboring E27K could precipitate the large-scale mtDNA deletions. Whereas current dogma holds that single large-scale mtDNA deletions are sporadic, our data now suggest that this SLSMD may have been mediated by dysfunctional SSBP1 harboring one or more mutant monomers.

The proband harbored a 5,440 base pair mtDNA deletion at 68% heteroplasmy in his fibroblast cell line and 16% heteroplasmy in blood, which is accepted as the immediate cause of his multi-system mitochondrial disorder. Because other deletions were not detected, this mtDNA deletion likely represents a clonally expanded deletion event that occurred early in the development of the proband. This deletion is unique and, unlike the common deletion[39], is not flanked by a direct repeat. Furthermore, no microhomology or secondary structure resides at the breakpoints to point to an obvious mechanism of deletion formation. It is entirely possible that other infrequent mtDNA deletions exist in this proband but at heteroplasmy levels too low (i.e., below one percent) to detect with current sequencing techniques. Deletions during replication can occur from DNA strand breaks or stalled replication forks, which can cause non-homologous rearrangement or replication strand slippage[40, 41]. SSBP1 functions during mtDNA replication to protect displaced ssDNA from damage, to prevent formation of DNA secondary structures, or to preclude the binding of inappropriate DNA metabolizing enzymes. In the strand displacement model of mtDNA synthesis, SSBP1 binds and protects the displaced heavy strand during light-strand-templated synthesis of the new heavy strand (Fig 1).

Biochemical assessments and molecular dynamics simulations ascertained the stability of SSBP1 tetramers with varying combinations of mutant and WT monomers. Biochemical analysis of the homotetrameric p.E27K SSBP1 revealed only modest differences in thermostability and DNA binding when compared with WT SSBP1. Simulated solution structures of mutant tetramers retained the β-barrel scaffold and exhibited relatively high dynamic characteristics for both the WT and p.E27K proteins, specifically in the β-hairpin and loop regions that are solvent exposed. Additionally, all mutant tetramers displayed slightly diminished mobility through their C_α_ deviation values when compared with WT SSBP1.

Because the proband harbors a heterozygous mutation, SSBP1 tetramers can include five different combinations of monomers, ranging from zero to four E27K monomers, and the heterotetramers can adopt a number of permutations in orientations of WT and mutant monomers. We carried out molecular modeling for representative tetramer variations for each of the five combinations (Fig 7**)**. Free energy calculations for the tetrameric combinations with one, two, three, or four E27K monomers/tetramer revealed more negative binding free energies (tighter binding) for certain inter-monomer interactions. The tetramers with the single mutant and the ab mutant showed the tightest binding, while the other three systems show only moderate changes. These moderate global changes reflect the averaging of larger local changes: strong and weak binding interactions among monomers are redistributed, compensating for one another. In the WT and various p.E27K tetramers, intra-dimer interactions (ab and cd) are the strongest. Interestingly, in every tetramer containing E27K monomers, some inter-dimer interactions are strengthened (ad and bc), indicating reduced molecular “breathing” within those tetramers relative to WT.

Taken together, the structural and biochemical information gathered in this study delineate a collection of subtle differences in structure and dynamics of the mutant SSBP1 protein. Based on the molecular dynamics simulations, it is important to consider how the composition and orientation of E27K monomers in the tetramer could affect the function of p.E27K SSBP1. Two possible mechanisms for the role of p.E27K SSBP1 in the introduction of a large mtDNA deletion can be supported by our findings. On one hand, an SSBP1 tetramer containing only one or two E27K monomers is predicted to have reduced positively-charged accessible surface area; this could result in looser ssDNA binding, leaving exposed ssDNA vulnerable during replication. On the other hand, an SSBP1 tetramer with three or four E27K monomers is predicted to have larger positively-charged accessible surface area and less molecular “breathing” caused by diminished mobility at the inter-dimer interface; these tetramers might bind ssDNA more tightly, slide along ssDNA with difficulty, or release ssDNA less readily, any of which could perturb normal function of the mtDNA replisome. Both mechanisms suggest plausible origins for the SLSMD, although each relies on different compositions of SSBP1 tetramers participating in creation of the initial deletion.

This report expands our understanding of the potential etiology of SLSMDs, suggesting that sporadic deletions may be caused by nuclear gene defects, changing the genetic counseling of recurrence risk in affected families and highlighting unique targets for potential future therapeutic development. Identification of a pathogenic variant in *SSBP1*, which is known to bind and protect ssDNA at the mitochondrial replication fork, further broadens understanding of mtDNA replication errors and expands the known molecular and phenotypic consequences of such errors. Whereas multiple mtDNA deletions have been previously associated with causal nuclear genetic etiologies, this is the first report of a SLSMD manifesting across the spectrum of classical clinical syndromes to result from a pathogenic variant in any nuclear gene. This discovery directly challenges the long-held assumption that SLSMDs are sporadic, and in most cases *de novo*, with low recurrence risk.

There are several likely reasons why nuclear genetic etiologies of SLSMDs have not been identified in the past. For individuals with features concerning for a primary mitochondrial disease, it has been standard clinical practice to end the search for an underlying molecular diagnosis when a SLSMD is found. Thus, high-throughput nuclear gene sequencing approaches such as WES would not have been clinically indicated nor pursued, and most individuals in whom a SLSMD was identified would not have undergone the type of comprehensive nuclear DNA gene analysis that has become clinically available in the last five to ten years. However, our work demonstrates that predisposition to SLSMDs may indeed follow an autosomal mode of inheritance. There have been scarce reports in the literature of SLSMDs being inherited and many clinicians do not test for the presence of the mtDNA deletion in mothers of affected individuals. If a mother is tested, analysis is likely only performed in an easily accessible tissue such as blood or saliva; however, SLSMDs are known to only occur in muscle in some individuals and may be eliminated by purifying selection in the most easily accessible tissues over time. Current technologies such as next generation sequencing and ddPCR can detect deletions present with heteroplasmy levels as low as 1–10% even in easily accessible tissues. This case demonstrates the need to pursue more sophisticated mtDNA sequencing and quantitative deletion analyses in suspected mitochondrial disease patients who have not received diagnostic testing performed by highly sensitive technologies.

The identification of a nuclear genetic etiology for a SLSMD directly impacts genetic counseling for individuals with SLSMDs. The proband reported here would have been counseled that recurrence risk for his own children was extremely low due to near-universal maternal inheritance of mtDNA through the oocyte in humans. Even if this proband were female, genetic counseling would have included a discussion of prior cases of maternal inheritance of SLSMDs, which is empirically estimated at approximately 4% inheritance risk[42]. Identification of a nuclear genetic etiology dramatically changes counseling for recurrence risk, as each future child of the proband has a 50% chance of inheriting the *SSBP1* pathogenic variant from him. Additionally, the risk of parental germline mosaicism for c.79G>A *SSBP1* must be considered in the recurrence risk estimation for any future children of the proband’s parents.

Our findings further highlight the significance of the surface patch in which E27, R38, and R107 reside. Another of the recently reported disease variants, S141N, is also found at the edge of this patch[21]. The clustering of disease variant residues in this patch is striking. As sequencing technology for clinical diagnosis expands and improves for mitochondrial disease patients, awareness of this patch and its constituent residues may help to identify additional SSBP1 variants.

In summary, we report here the first nuclear gene defect associated with a SLSMD in a proband with severe, progressive, multi-system manifestations of all classical SLSMD clinical syndromes, namely Pearson syndrome, Kearns-Sayre syndrome, and Leigh syndrome. The *de novo* heterozygous missense variant c.79G>A in the nuclear gene *SSBP1* results in an E27K substitution in the SSBP1 protein. Co-expression of WT and p.E27K SSBP1 monomers will produce a mixed population of SSBP1 tetramers—we have shown that SSBP1 tetramers containing p.E27K monomers have different physical properties from WT tetramers, which may alter ssDNA binding. Altered ssDNA binding in mitochondria could directly interfere with proper mtDNA replication. Compromised mtDNA replication is consistent with generation of a SLSMD, as was observed in the mitochondrial disease proband. Contrary to long-held dogma that single large-scale mtDNA deletions are sporadic, this work suggests that SLSMDs may result from nuclear gene disorders that disrupt mtDNA replication.

## Supporting information

S1 File Proband clinical and laboratory testing detailed description. (DOC)

S1 Table Binding free energy (kcal/mol). Free energies were calculated for each selected monomer pair indicated at the top. Free energies calculated using the MMPBSA module utilized the coordinates of each tetramer from the last 100 ns selected at each nanosecond. Monomers underlined are E27K. Highlight colors reflect differences from the WT tetramer in each comparison: yellow indicates a less negative value (looser binding); blue indicates a more negative value (tighter binding).
(XLSX)

S1 Fig Brain MRI performed in the proband at age 10.5 years. **A.** Sagittal T1 demonstrates cerebellar atrophy, with prominence of the folia (thin arrow), thinning of the corpus callosum (star) and a retrocerebellar cyst (thick arrow). **B.** Axial T2 demonstrates hyperintensities in bilateral globus palladi (right>left, thin arrows), thalami (left>right, thick arrows), multiple lesions in posterior corpus callosum (ray), ventriculomegaly and cortical atrophy, and posterior white matter hyperintensities (right>left, stars). **C.** Axial T2 demonstrates bilateral substantia nigral hyperintensities (arrows). **D.** Axial T2 demonstrates midbrain hyperintense lesions (arrows).
(TIF)

S2 Fig Retinal imaging. **A.** Composite fundus picture of left eye (LE) at age 11. Note greyish hue of extensive outer retinal atrophy in retinal mid- and far periphery; because of atrophy of outer retinal layers, choroidal vessels are better visible; white veils in retina represent prominent subretinal fibrosis, more pronounced in nasal midperiphery; moderate attenuation of retinal vasculature; both subretinal fibrosis and vascular attenuation are secondary to progressive retinal dystrophy; no intraretinal pigment migration of note as yet. **B.** Vertical optical coherence tomography (OCT) scan of central macula of left eye (LE) at age 11. Note preservation of outer retinal layers representing photoreceptors and retinal pigment epithelium only in central macula, in and immediately around fovea; extreme paucity of cells beyond that central area, in keeping with completely abolished gross rod and cone function on full-field flash electroretinography. Total surface area of remaining functioning retina is too small to be measurable on ERG.
(TIF)

S3 Fig Audiogram. Audiology evaluation at age 12 years showing bilateral high frequency mild to moderate-severe sensorineural hearing loss.
(TIF)

S4 Fig Growth parameters. A. Height. B. Body mass index (BMI). C. Weight. Blue circles depict clinical measurements of the proband.
(TIF)

S5 Fig Analysis of proband and parental cells. A. Western blot of SSBP1 protein in proband and parent fibroblast and lymphoblastoid cell lines with β-actin loading control (left panel). Quantitation of the western blot with proband signal normalized to parent signal for each cell type (right panel). B. Mitochondrial respiratory capacity as measured by Oroboros in proband and parent cells. Error bars represent SEM; n = 3 C. mtDNA copy number in proband and parent cells as measured by real-time PCR using oligonucleotide probes against the mitochondrial *ND1* gene (left graph) and the mitochondrial *COX3* gene (right graph).
(TIF)

S6 Fig A single SSBP1 tetramer, WT or p.E27K, binds each molecule of ssDNA substrate used for fluorescence anisotropy assays. Binding reactions contained 30 mM HEPES-KOH pH 7.6, 50 mM KCl, 2 mM dithiothreitol, 10% glycerol, 20 nm FAM-labeled 50 nucleotide ssDNA substrate, and either no SSBP1 or 20 nm (tetramer) WT or p.E27K SSBP1. Samples were resolved on an 8% polyacrylamide gel in 1X TBE. Electrophoretic mobility shift images were collected on a Typhoon FLA 9500 with a 473 nm excitation laser and LBP filter. The mobilities of unbound and bound DNA species are indicated. All lanes presented were run on one gel, cropped for clarity.
(TIF)

S7 Fig Convergence of root mean square deviation (RMSD) values confirms that each system has achieved stability during the trajectory calculation. From the coordinates selected at a nanosecond interval along the trajectory, RMSD values were calculated for backbone heavy atoms using the coordinates of the X-ray crystal of SSBP1 as the reference structure. WT monomer and dimer systems were simulated under the same conditions as tetramers to create references for the stability of monomer and dimer conformations. In the inserted legend, letters in the parenthesis represent the labels of mutated monomers.
(TIF)

S8 Fig Normalized B-factors reveal significant position fluctuations in loop regions. The six configurations simulated are shown (**A-F**). Residue based calculations were carried out for the structures extracted from the last 100 ns of each MD trajectory, and averaged values are displayed. B-factors calculated from monomer and dimer simulations are also displayed for comparison.
(TIF)

S9 Fig Electrostatic surface potentials reveal that long patches of positive charge are available for interactions with negatively charged ssDNA. The six configurations simulated are shown (**A-F**). Modeled solution structures are shown as surface models colored for electrostatic surface potential. Regions in blue are positively charged; regions in red are negatively charged.
(TIF)
