# Supplementary material for: Mitochondrial single-stranded DNA binding protein novel de novo SSBP1 mutation in a child with single large-scale mtDNA deletion (SLSMD) clinically manifesting as Pearson, Kearns-Sayre, and Leigh syndromes
Source: PLoS One. 2019 Sep 3;14(9):e0221829. doi: 10.1371/journal.pone.0221829 (PMC6719858; doi:10.1371/journal.pone.0221829)
Supplement: S1 File — (DOC) [file pone.0221829.s001.doc]

**SUPPLEMENTAL FILES.**

**Supplemental File 1. Proband Clinical and Laboratory Testing Detailed Description.**

Proband – Detailed Clinical Description.

*Birth History*. The proband is a Chinese boy born at full-term following an unremarkable prenatal history by emergency cesarean section due to fetal bradycardia. Birth weight was 6 pounds, 11 ounces and length was 20 inches. There were no neonatal complications. Family history was non-contributory, with no significant medical problems.

*Developmental history*. The proband had early motor development with independent walking at 10 months and was toilet trained by age 2.5 years. However, he had early speech delay, with babbling at 1.5 years old and not speaking meaningful words until age 2 years. He had no history of early developmental regression. His family noticed it has always taken him longer than others to learn new things like numbers or ABCs. Learning difficulties became more apparent when he started kindergarten around age 5 years, as he was unable to keep up with his peers academically. Currently, he is in a specialized classroom (small class size with three teachers). His reading ability in the third grade was found to be advanced at a fifth grade reading level, but with problems with reading speed and comprehension. He loves reading and at age 14 years can still read large print books. IQ testing at approximately age 10 years was reportedly normal, although no formal report is available for review.

*Infantile Anemia and Bone Marrow Failure*. He was healthy until age 5 months when he was noted to be pale and listless, and found to have pancytopenia. He was diagnosed with severe aplastic anemia and underwent multiple blood transfusions.

Bone marrow biopsy at age 9 months was consistent with a bone marrow failure syndrome, showing markedly hypocellular

marrow (5%) and decreased number of myeloid cells, erythroid cells and megakaryocytes with maturation, but no tumor,

lymphoid aggregate, or granuloma. Bone marrow aspirate showed relative erythroid hypoplasia, cytoplasmic to nuclear maturation asynchrony in the myeloid precursor cells, occasional vacuolated blasts, and chemical staining was normal with no ringed sideroblasts seen on iron staining. Immune suppression therapy began with antithymocyte globulin and cyclosporine at age one year, with resulting increase in hemoglobin levels. Therapy ended at age 2.5 years, and he has remained mildly anemic.

*Growth Failure.* While always small compared to his peers, his poor growth became apparent at age 5 years. Always a slow and picky eater, multiple nutritional interventions including appetite stimulant and emphasis on nutrition were employed without success. Despite a mid-parental height of ~10th percentile, height percentile fell from 33rd percentile (Z- score, -0.43) at age 3 years, to 2nd percentile (Z-score, -2.05) at age 5 years. He underwent growth hormone screening at age 7 years, which identified peak growth hormone response of 9.08 ng/mL (borderline low), normal pituitary MRI, and low growth factors (at age six years IGF-1 level was 70 ng/mL, mean 144 and IGF-BP3 was 1.6 mg/L, mean 2.4; at age seven years IGF-1 level was 69 ng/mL, mean 187 and IGF-BP3 was 2.0 mg/L, mean 3), which raised concern for growth hormone deficiency. From age 8 years to 11 years he was treated with growth hormone in hopes of achieving some growth benefit; it was discontinued for concerns regarding lack of efficacy and potential side effects. Bone age was not delayed. By age 12 years, height percentile fell further to <1st percentile (Z-score, -4.55), and low-dose growth hormone was restarted in attempt to improve decreased muscle mass and risk for low bone mineral density, although height-adjusted aBMD was not significantly low. Currently at age 14 years his height is at the 3rd percentile (Z score = -4.85; average height of a 7-year- old). Given concern for failure-to-thrive (BMI plotting to the <3rd percentile), decreased oral intake and dehydration, renal disease, and choking, gastrostomy tube was placed at age 12 years. He had difficulty tolerating feeds, which raised concern for gastroparesis but gastric emptying scan was deferred by the family. He started erythromycin at age 14 years, which led to decreased vomiting and some weight gain (2.5 pounds) after 4 weeks on treatment.

*Ophthalmologic Problems.* Between the ages of 4-5 years, he began complaining of having trouble seeing objects at distance and at night. He was subsequently diagnosed with astigmatism, and bilateral myopic and astigmatic glasses were prescribed. Bilateral ptosis was noted at age 9 years. By 10 years old, he had bilateral ophthalmoplegia. At age 11 years, he underwent detailed ophthalmologic and retinal evaluation. By this time, he had complete nyctalopia, concentrically constricted visual fields, and low best-corrected visual acuity (20/125 OD and 20/125 OS). His fundus exam was significant for retinal vessel attenuation and extensive outer retinal atrophy associated with subretinal fibrosis in the midperiphery (Supplemental Figure 2A). Full-field electroretinography showed absent rod-specific, combined rod-cone, and cone-specific responses bilaterally, consistent with severe rod-cone dystrophy. Eye movements were nearly completely absent due to external ophthalmoplegia. Recently, at age 14 years, his best-corrected visual acuity was only at 20/100 in the right eye and 20/600 in the left eye, due to further loss of central macular structure and function (Supplemental Figure 2B). He was also found to have inferior corneal infiltrates and early corneal ulcer formation due to chronic corneal exposure. This is because of his incompletely closed eyelids during the night, despite continuing ptosis, and absent Bell phenomenon consequent upon external ophthalmoplegia with absent upgaze.

*Endocrine Problems. (1) Diabetes Mellitus.* After ~three months on low-dose growth hormone therapy, he had an elevated HbA1C (7%), prompting admission. Of note, 1 out of 3 pancreatic auto-antibodies was mildly positive (2.2 U/mL, <1.0). His insulin requirement was mainly to cover overnight feeds; he responded very well to a low dose of insulin. Growth hormone was discontinued, as were overnight feeds, and he no longer required insulin. At age 14 years, he was again started on tube feeds, which was associated with the development of polyuria, polydipsia, and increased blood glucose, again requiring insulin. Pump therapy is being pursued. (2) *Hypothyroidism*. He had central hypothyroidism treated with levothyroxine since age 7.5 years. (3) *Dysautonomia*. He was found to have decreased sweating and heat intolerance noted after cyclosporine treatment. He persistently feels cold. (4) *Risk for other endocrinopathies.* Repeated surveillance studies for adrenal insufficiency and hypoparathyroidism were reassuring. Vitamin D deficiency was treated with vitamin D3.

*Neurologic Problems.* He has congenital hypotonia, lifelong fatigue, and exercise intolerance. Over time, he has had progressive regression in his gross and fine motor skills. By age 12 years, he preferred to crawl rather than walk because he was afraid of tripping and falling due to his progressive ataxia. He started to rely on holding onto furniture to move around the house. Currently at 14 years old, he continues to have a progressive movement disorder, including a cerebellar syndrome. He has dysarthria as well as a tremor which affects his voice, and his family has increasing difficulty understanding him. He has central titubation of the neck and body, and dysmetria of the bilateral arms. His handwriting has deteriorated due to tremor, dysmetria and dyspraxia. He has poor aim with urination. He can crawl up and down stairs with supervision but is no longer able to walk independently, due to proximal weakness, central titubation, and ataxia, and is reliant on his walker to get around or holding someone’s hand. Brain MRI at age 10.5 years (Supplemental Figure 1) showed findings consistent

with Leigh syndrome, including bilateral symmetric hypointense T1 and hyperintense T2 signal of bilateral globus pallidi

and thalami, extending into each cerebral peduncle, substantia nigra, midbrain (affecting dorsal nuclei including medial

longitudinal fasciculus and abducens nuclei, and tectal nuclei), and lower pons. More subtle abnormal FLAIR and T2 signal

were seen within the frontal and parietal cerebral hemispheres. He has not undergone repeat brain MRI. MRS was not

performed. Nerve conduction studies (NCS) and electromyogram (EMG) were performed at age 11 years. NCS was normal. EMG identified reduced activation in multiple muscles, consistent with central nervous system pathology.

*Sensorineural Hearing Loss.* High frequency mild to moderate-severe sensorineural hearing loss was detected at age 12 years. He has not undergone a follow-up evaluation, although his family reports they have had to speak louder and louder to him over time.

*Chronic kidney disease*. He had normal kidney function until age 8 years at which point he developed persistently elevated serum creatinine to 0.6 mg/dL, corresponding to a creatinine-estimated glomerular filtration rate (GFR) of 75 ml/min/1.73m2 or stage II chronic kidney disease (CKD) (normal GFR >90 ml/min/1.73m^2^). Over the next six years, his kidney function has progressively worsened to stage V CKD with a serum creatinine of 3.7 mg/dL, corresponding to a GFR of 14 ml/min/1.73m^2^. Due to his very low muscle mass, it is possible that his serum creatinine overestimates his kidney function and that his actual GFR is even lower. He has required hospitalization for hyperkalemia and acute elevations in creatinine that have responded to fluid administration. Although he has intrinsic CKD from his mitochondrial disease, his chronic decreased fluid intake, failure to thrive, and polyuria from diabetes mellitus may also contribute to a pre-renal component

of his elevated creatinine. Renal ultrasound has shown diffuse bilateral increased renal cortical echogenicity consistent with

medical renal disease without urinary tract dilatation. Blood pressures have been intermittently and mildly elevated and

urinalyses have been without significant proteinuria. Despite his severe CKD, he has normal potassium, no persistent

metabolic acidosis, no hyperphosphatemia or hypocalcemia, and no secondary hyperparathyroidism. He has mild anemia,

but this could be related to his underlying condition. His parents have not been interested in pursuing options for renal

replacement therapy (dialysis or kidney transplant) at this time.

*Cardiac Problems.* Cardiac evaluation at age 11 years was normal except for his aortic root diameter being top normal and mildly increased pulmonary artery for body surface area. At age 12 years, he was found to have right bundle branch block. 24-hour Holter monitoring identified occasional atrial and ventricular ectopy. Repeat 24-hour Holter monitoring at age 14 years revealed occasional premature atrial contractions, bundle branch block, and prolonged PR. His family has declined pacemaker placement.

Proband Laboratory Testing.

*Metabolic Screening Studies*. Comprehensive metabolic screening studies have been performed and revealed mild lactic acidemia (ranging from 2.43 mM to 4.74; normal 0.8-2.0 mM); elevated lactate to pyruvate ratios (ranging from 22 to 40; normal 10-20); elevated acylcarnitines including glutarylcarnitine; normal total and free carnitine analyses; and normal urine organic analyses. Plasma amino acid analyses were normal with the exception of elevated alanine on two occasions (575.2 and 646.3 µmol/L; normal 89-440); elevated alanine to phenylalanine and tyrosine ratios (4.13, 5.3, 7.4), and elevated alanine to lysine ratio (3.21, 3.4, 4.1). Lipoprotein panels have been normal. Cerebrospinal fluid (CSF) analysis at age 10 years identified increased red blood cells (1 CUMM, normal 0), increased total protein (171 mg/dL; normal 15-40), normal neurotransmitters, elevated CSF lactate (4.34 mM; normal 0.7-2.0), and elevated lactate to pyruvate ratio (33; normal 10- 20)

*Clinical Diagnostic Genetic Testing*. Molecular diagnostic evaluation for aplastic anemia was performed over time and was normal, including *SBDS* sequencing, telomere length analysis, chromosome breakage and radial forms analysis in blood,

sequencing of *TERC*, *DKC1, GATA1*, *RMRP, RPL5*, *RPS19*, *RPL11*, *RPS19,* as well as *RPS19* deletion and duplication

analysis. Genome-wide SNP microarray analysis was normal, with no chromosomal copy number alterations identified. A combined next generation sequencing panel of 139 nuclear genes and the mtDNA genome performed at GeneDx in blood at age 11 years identified a 5 kilobase deletion (m.8629_14068del5440) reported to be present at less than 15% heteroplasmy. Several heterozygous single variants were identified in nuclear genes associated with autosomal recessive disorders although no second variant was detected on the other allele. Clinical exome sequencing by next generation sequencing of the nuclear genome and mtDNA genome performed at age 12 years in blood from the proband (with exome also performed in both parents but mtDNA sequencing not performed in the mother’s blood) again identified the 5 kilobase deletion (m.8629_14068del5440), estimated to be present at less than 15% heteroplasmy in the proband. No clinically significant pathogenic variants in nuclear genes associated with mitochondrial disease were reported, although a *de novo* variant was identified in a gene without a known disease association at the time (c.79G>A: p.E27K), *SSBP1*.
