# Supplementary figures and images for: Mitochondrial single-stranded DNA binding protein novel de novo SSBP1 mutation in a child with single large-scale mtDNA deletion (SLSMD) clinically manifesting as Pearson, Kearns-Sayre, and Leigh syndromes
Source: PLoS One. 2019 Sep 3;14(9):e0221829. doi: 10.1371/journal.pone.0221829 (PMC6719858; doi:10.1371/journal.pone.0221829)

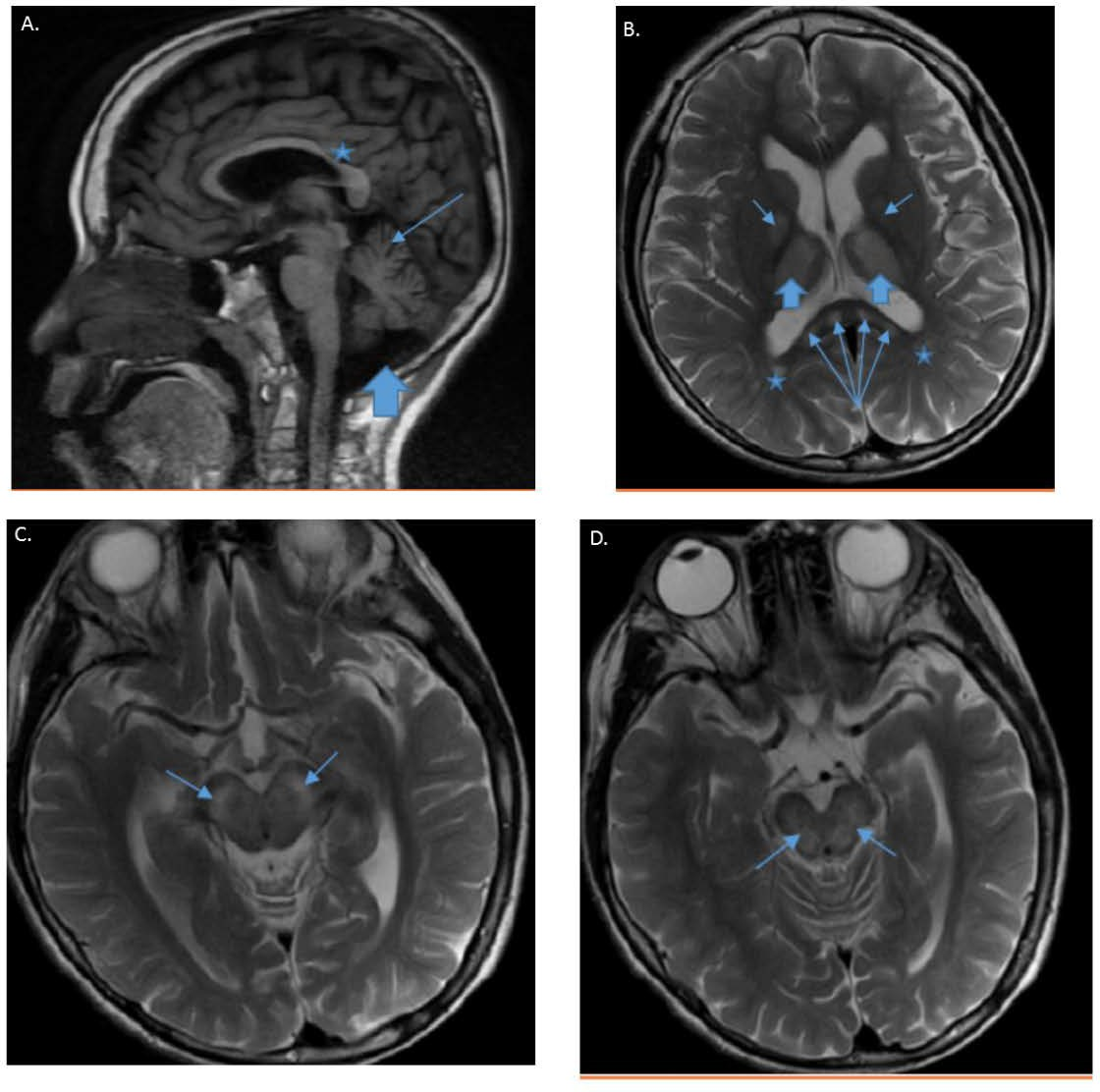

Supplement: S1 Fig — A. Sagittal T1 demonstrates cerebellar atrophy, with prominence of the folia (thin arrow), thinning of the corpus callosum (star) and a retrocerebellar cyst (thick arrow). B. Axial T2 demonstrates hyperintensities in bilateral globus palladi (right>left, thin arrows), thalami (left>right, thick arrows), multiple lesions in posterior corpus callosum (ray), ventriculomegaly and cortical atrophy, and posterior white matter hyperintensities (right>left, stars). C. Axial T2 demonstrates bilateral substantia nigral hyperintensities (arrows). D. Axial T2 demonstrates midbrain hyperintense lesions (arrows). (TIF) [file pone.0221829.s003.tif]

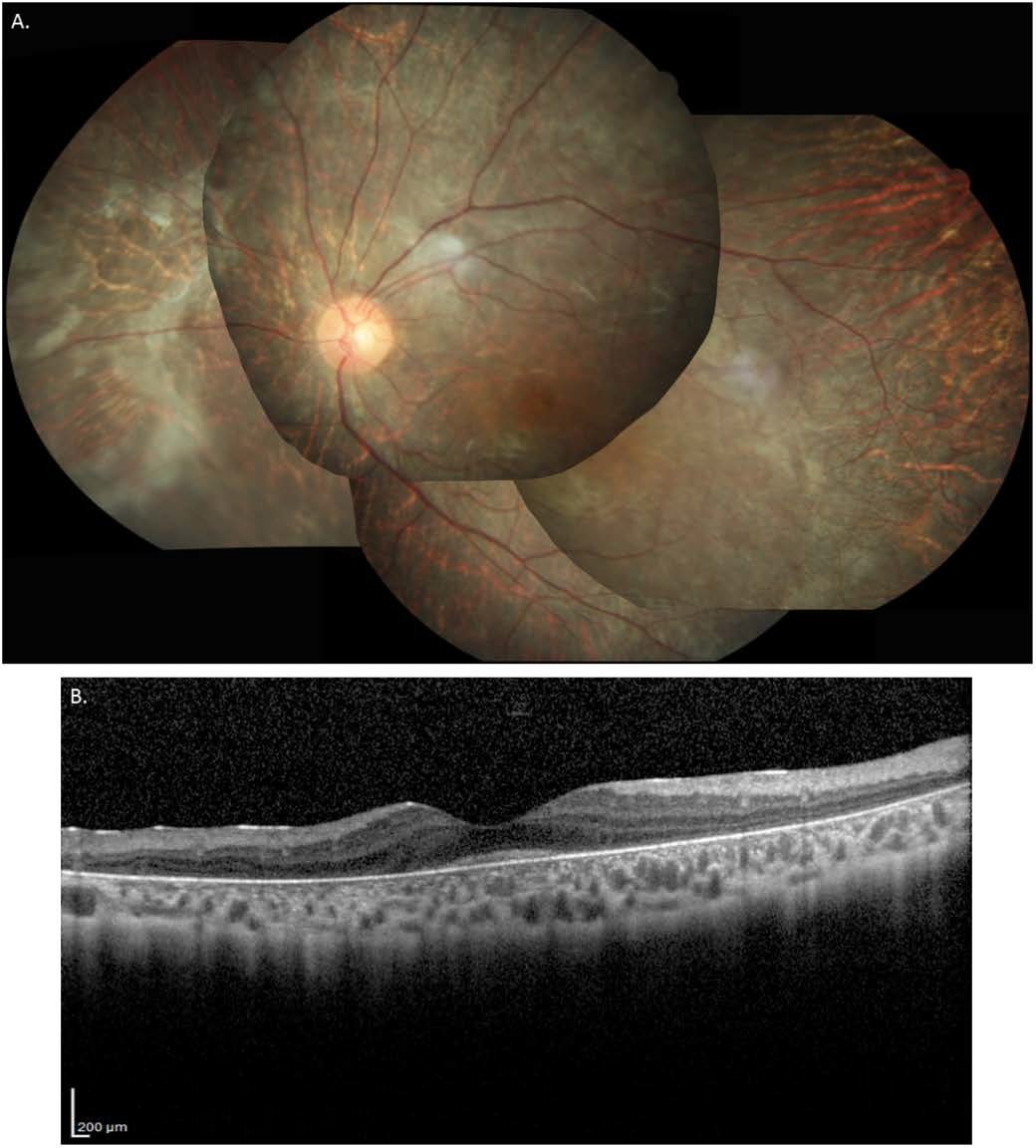

Supplement: S2 Fig — A. Composite fundus picture of left eye (LE) at age 11. Note greyish hue of extensive outer retinal atrophy in retinal mid- and far periphery; because of atrophy of outer retinal layers, choroidal vessels are better visible; white veils in retina represent prominent subretinal fibrosis, more pronounced in nasal midperiphery; moderate attenuation of retinal vasculature; both subretinal fibrosis and vascular attenuation are secondary to progressive retinal dystrophy; no intraretinal pigment migration of note as yet. B. Vertical optical coherence tomography (OCT) scan of central macula of left eye (LE) at age 11. Note preservation of outer retinal layers representing photoreceptors and retinal pigment epithelium only in central macula, in and immediately around fovea; extreme paucity of cells beyond that central area, in keeping with completely abolished gross rod and cone function on full-field flash electroretinography. Total surface area of remaining functioning retina is too small to be measurable on ERG. (TIF) [file pone.0221829.s004.tif]

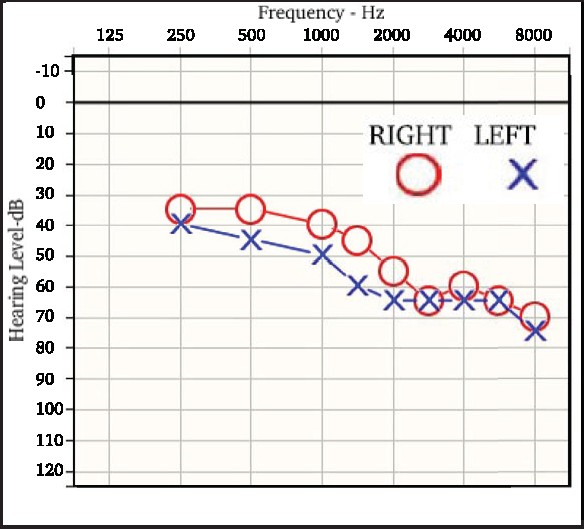

Supplement: S3 Fig — Audiology evaluation at age 12 years showing bilateral high frequency mild to moderate-severe sensorineural hearing loss. (TIF) [file pone.0221829.s005.tif]

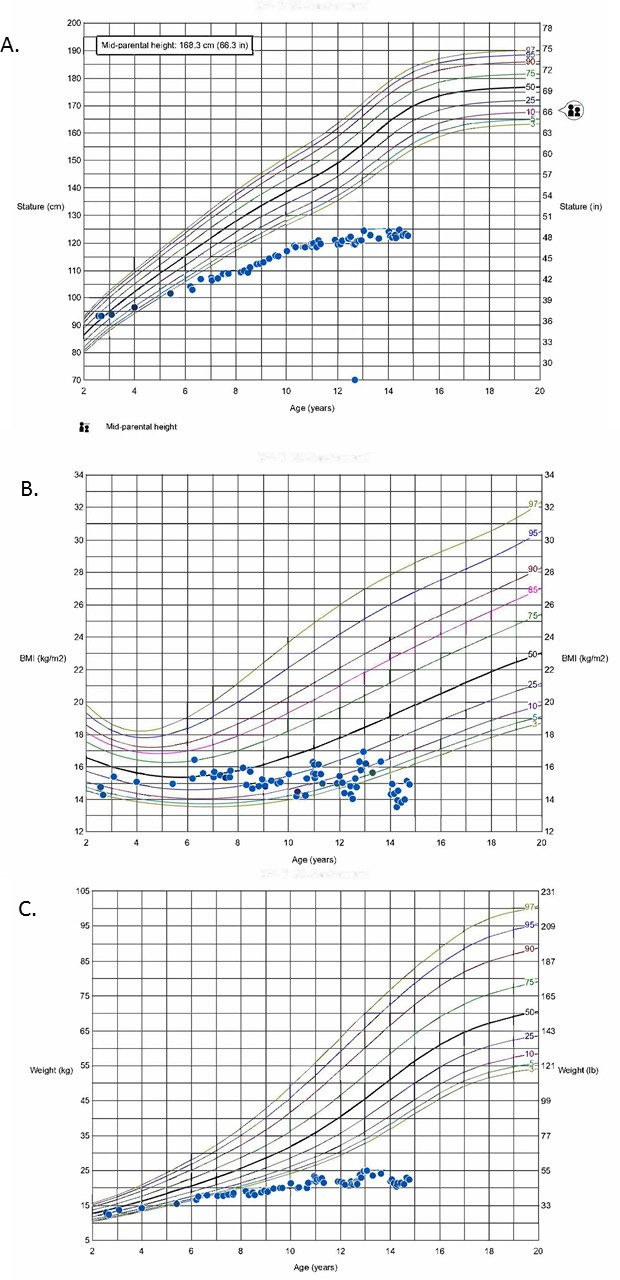

Supplement: S4 Fig — A. Height. B. Body mass index (BMI). C. Weight. Blue circles depict clinical measurements of the proband. (TIF) [file pone.0221829.s006.tif]

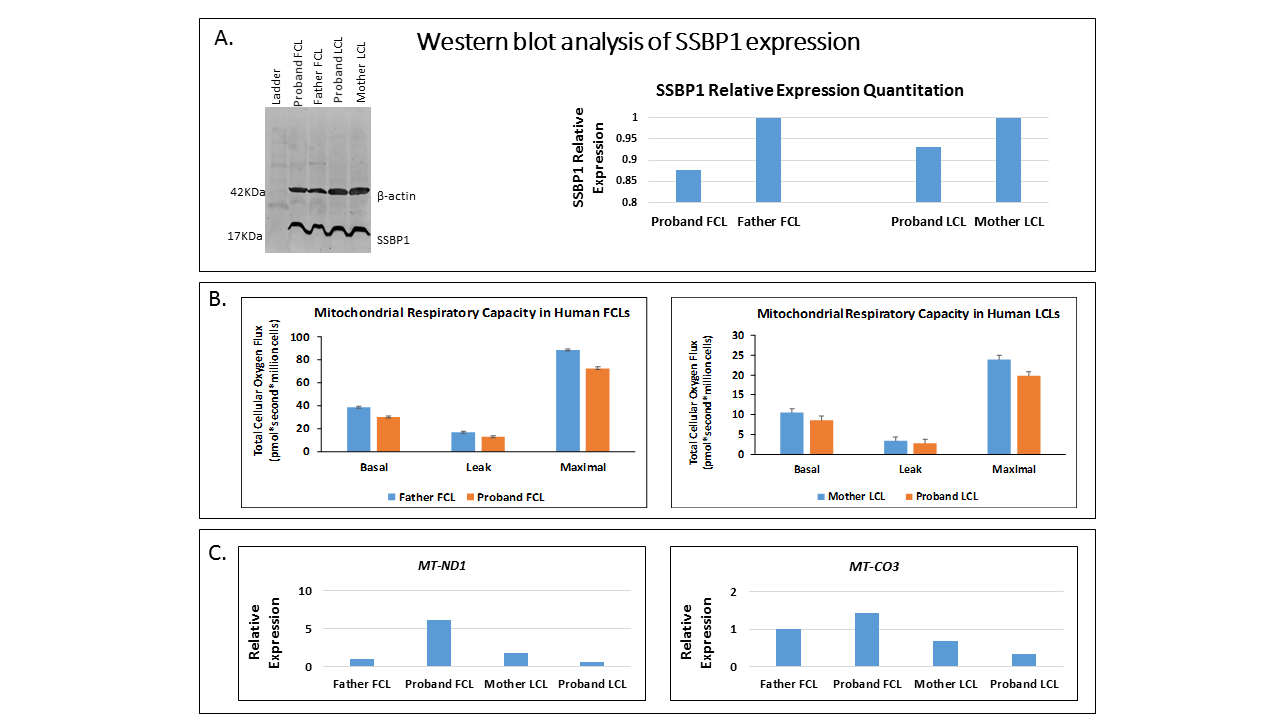

Supplement: S5 Fig — A. Western blot of SSBP1 protein in proband and parent fibroblast and lymphoblastoid cell lines with β-actin loading control (left panel). Quantitation of the western blot with proband signal normalized to parent signal for each cell type (right panel). B. Mitochondrial respiratory capacity as measured by Oroboros in proband and parent cells. Error bars represent SEM; n = 3 C. mtDNA copy number in proband and parent cells as measured by real-time PCR using oligonucleotide probes against the mitochondrial ND1 gene (left graph) and the mitochondrial COX3 gene (right graph). (TIF) [file pone.0221829.s007.tif]

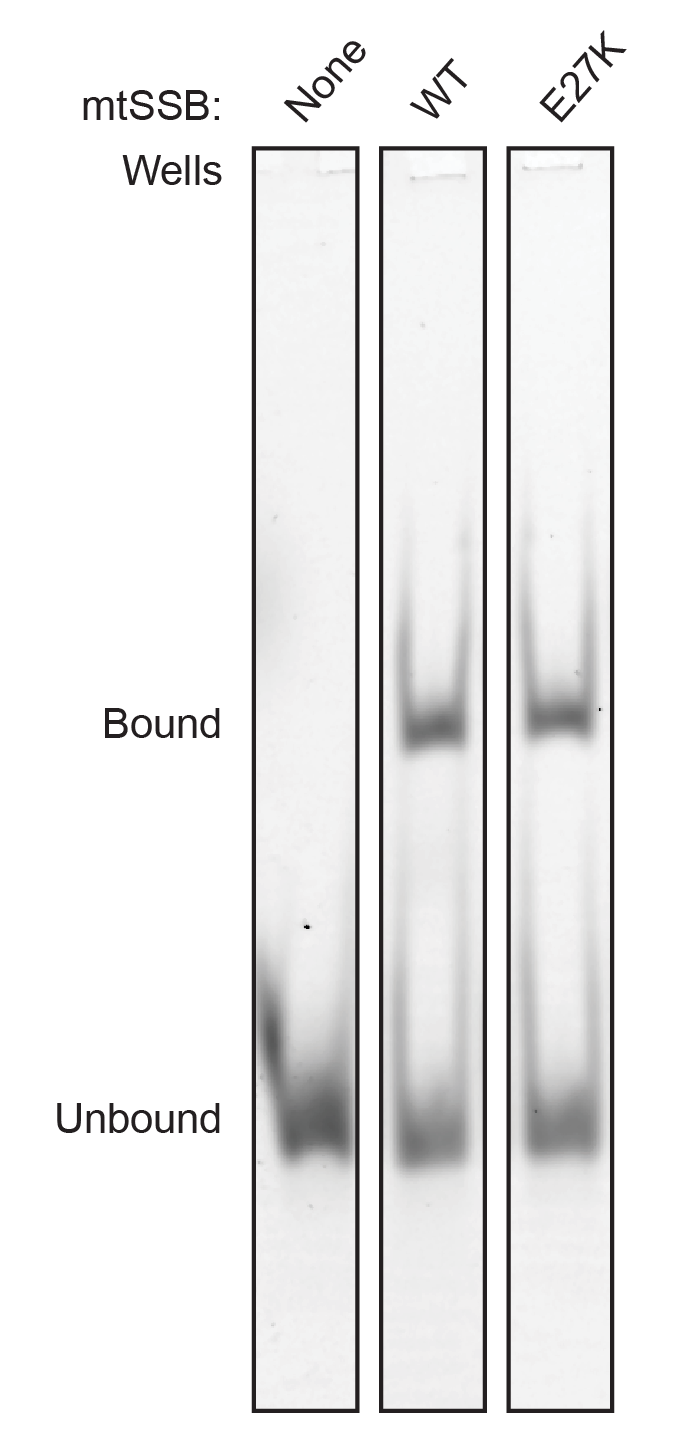

Supplement: S6 Fig — Binding reactions contained 30 mM HEPES-KOH pH 7.6, 50 mM KCl, 2 mM dithiothreitol, 10% glycerol, 20 nm FAM-labeled 50 nucleotide ssDNA substrate, and either no SSBP1 or 20 nm (tetramer) WT or p.E27K SSBP1. Samples were resolved on an 8% polyacrylamide gel in 1X TBE. Electrophoretic mobility shift images were collected on a Typhoon FLA 9500 with a 473 nm excitation laser and LBP filter. The mobilities of unbound and bound DNA species are indicated. All lanes presented were run on one gel, cropped for clarity. (TIF) [file pone.0221829.s008.tif]

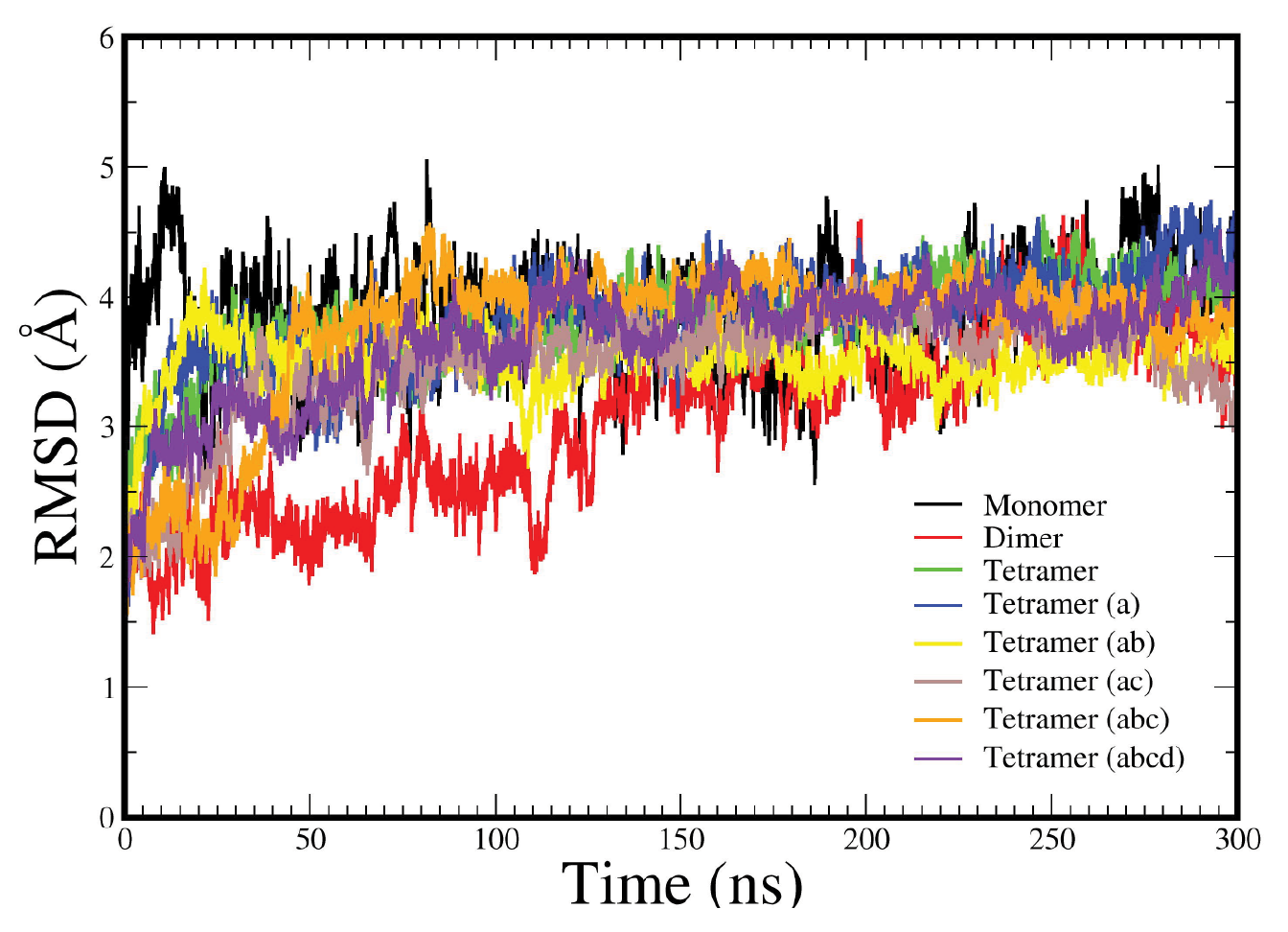

Supplement: S7 Fig — From the coordinates selected at a nanosecond interval along the trajectory, RMSD values were calculated for backbone heavy atoms using the coordinates of the X-ray crystal of SSBP1 as the reference structure. WT monomer and dimer systems were simulated under the same conditions as tetramers to create references for the stability of monomer and dimer conformations. In the inserted legend, letters in the parenthesis represent the labels of mutated monomers. (TIF) [file pone.0221829.s009.tif]

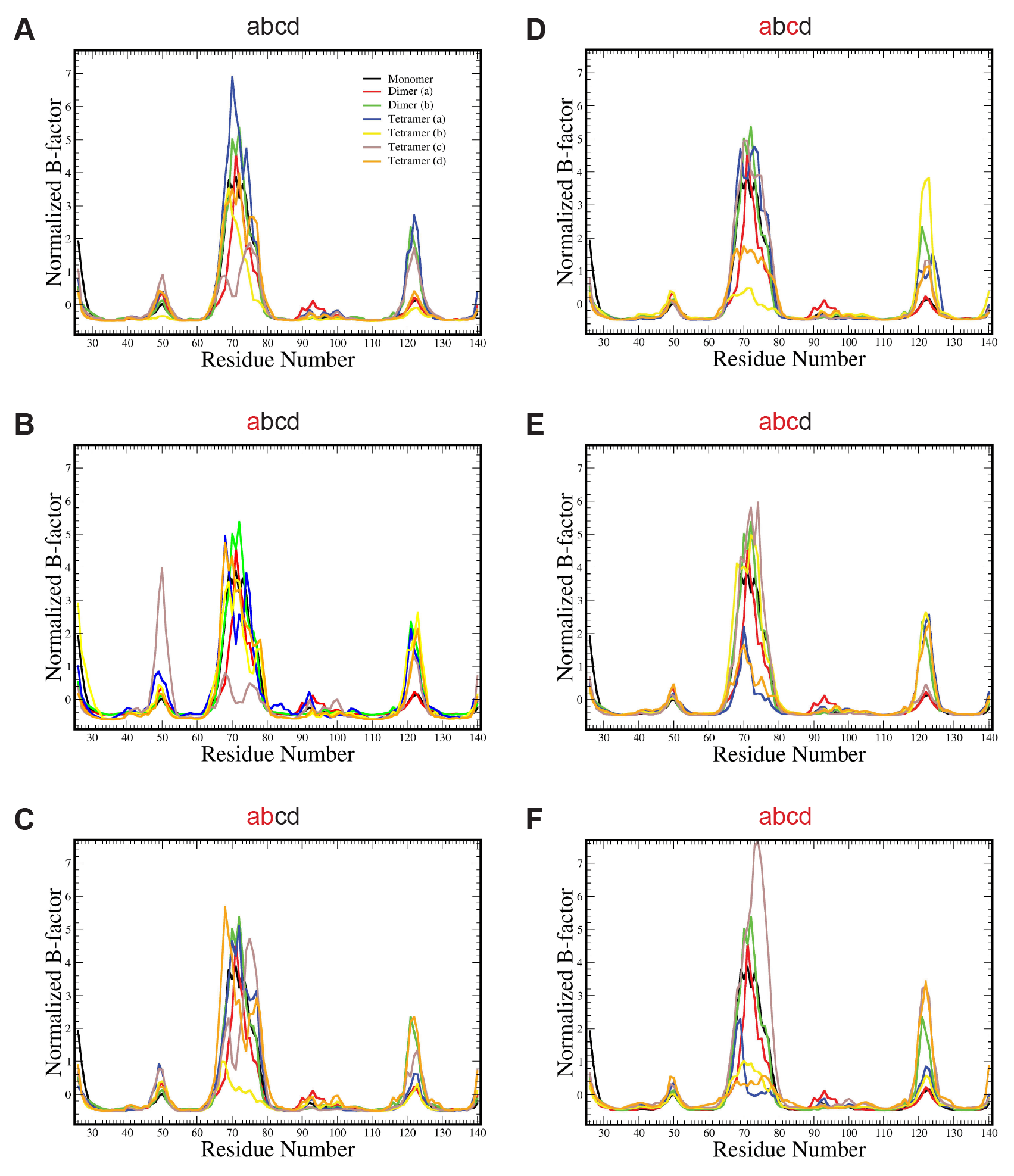

Supplement: S8 Fig — The six configurations simulated are shown (A-F). Residue based calculations were carried out for the structures extracted from the last 100 ns of each MD trajectory, and averaged values are displayed. B-factors calculated from monomer and dimer simulations are also displayed for comparison. (TIF) [file pone.0221829.s010.tif]

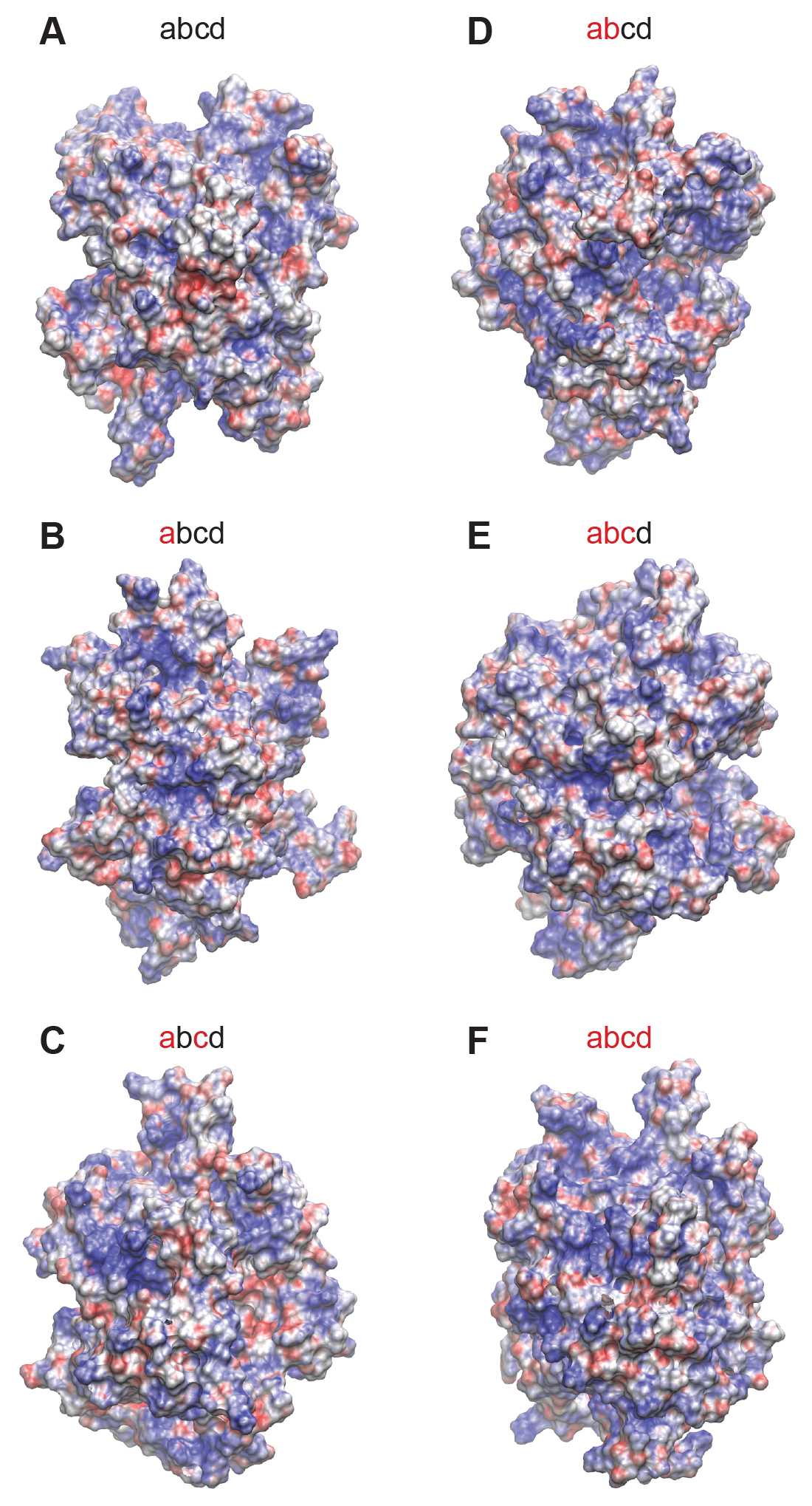

Supplement: S9 Fig — The six configurations simulated are shown (A-F). Modeled solution structures are shown as surface models colored for electrostatic surface potential. Regions in blue are positively charged; regions in red are negatively charged. (TIF) [file pone.0221829.s011.tif]
